# Ambient temperature influenced co-expression network of major developmental, circadian, and photoreceptor genes in bread wheat

**DOI:** 10.1038/s41598-025-12424-6

**Published:** 2025-07-30

**Authors:** Tibor Kiss, Ádam D. Horváth, András Cseh, Zita Berki, Krisztina Balla, Marianna Mayer, Viola Tóth, Ildikó Karsai

**Affiliations:** 1 HUN-REN Centre for Agricultural Research, Agricultural Institute, 2462 Martonvásár, Hungary; 2 Food and Wine Research Institute, Eszterházy Károly Catholic University, 3300 Eger, Hungary

**Keywords:** Vernalization, Photoperiod, Circadian and photoreceptor genes, Daily gene transcription, Gene network system, Hexaploid wheat, Molecular biology, Plant sciences, Environmental sciences

## Abstract

The developmental process of bread wheat comprises of two major phases: the generative development of the apices from double ridge to terminal spikelet formation, followed by the intensive stem elongation. The two phases differ significantly in terms of the most influential environmental stimuli; ambient temperature above the vernalization threshold exert a more pronounced influence on the molecular-genetic regulation of intensive stem elongation. We assume that dynamic interactions among circadian rhythms, photoreceptors, and key developmental genes play a critical role in shaping the genotypic responses. For this purpose, we chose three, well characterised winter bread wheat varieties with different genetic backgrounds and developmental patterns, in which we studied the daily expression of main developmental (*VRN1*, *VRN2*, *VRN3, PPD1*), circadian (*CCA1*, *PRR95*, *TOC1*, *LUX*, *ELF3*, *GI*, *CO1*) and photoreceptor (*PHYA*, *PHYB*, *PHYC*, *CRY1*, *CRY2*) genes using generic primers and determined their possible relationship under three environments (18 °C vernalized/unvernalized and 25 °C vernalized in the phytotron). The correlation-based network analyses underlined the strong probability of several gene interactions. The positive relationship between *VRN1* and *VRN3* existed in all treatments confirming that the close relationship between these two genes is essential for the flowering regulation. The vernalized *VRN2* showed an explicit diurnal activity in late heading cultivars, which became most expressive at 18 °C. In vernalized plants at 18 °C, *PPD1* expression was significantly increased in all three cultivars, becoming more pronounced in late heading cultivars. We found a significant negative association between *CCA1* and *TOC1*, in addition a significant negative association between *CCA1* and *LUX* and a significant positive correlation between *TOC1* and *LUX* was observed, irrespective to the environment. The close temperature-independent relationship between these major circadian genes may also illustrate their fundamental role in the floral regulatory system. Another strong positive correlation was observed between *GI* vs *LUX* and *PHYC* vs *ELF3*, independently of the environment. Our results, obtained by studying gene expression patterns within the complexity of whole-genome backgrounds, provide complementary information to the knowledge derived from studies using mutant and/or near-isogenic lines. They demonstrate the environmentally driven genetic plasticity present in varieties in response to diverse environmental cues, which may represent an important factor in ecological adaptation and a key element in improving resilience to climate change.

**Supplementary Information:**

The online version contains supplementary material available at 10.1038/s41598-025-12424-6.

## Introduction

The genetic regulation of plant development and flowering has been dissected in the model plant *Arabidopsis* to the largest extent, revealing the complex intricate system that enables the plants to percept the various environmental signals and to adjust their development accordingly. The large amount of information collected in *Arabidopsis* contributed greatly to the dissection and to the better understanding of this regulation network in cultivated plant species^1^. In temperate small grain cereals, it helped to identify various genes of the vernalization and photoperiod response pathways, several members of the circadian clock (input, core oscillation and output genes), photoreceptors, plant hormonal pathways, and central elements of flower initiation, with new findings published almost daily. These comparative studies have revealed similarities and basic differences between *Arabidopsis* and cereals in the structures and functionality of orthologous genes and in the compositions of the specific regulation pathways.

Due to these studies, the basic features of the two most important regulation pathways in cereals—vernalization and photoperiod response—have been fairly deeply dissected. The vernalization requirement is determined by the epistatic interactions between two major vernalization response gene families. These are the dominant flowering activators *VRN1* (orthologue of the *Arabidopsis* gene *AP1*) and the dominant flowering repressors *VRN2* (*ZCCT*, having no orthologue in *Arabidopsis*)^2–11^. According to the latest flowering regulation models of cereals, in autumn, after germination, when the days are still sufficiently long, the active *VRN2* gene prevents the transcription of the *VRN3* gene (orthologue of the *Arabidopsis* gene *FT*), which could be important in maintaining the activity of *VRN1* at a low level^12,13^. In the course of vernalization, the activity of the *VRN1* gene is gradually enhanced by the cold effect, leading to increasing expression of VRN1 transcription factors, which inhibit the functioning of *VRN2* while stimulating that of *VRN3*^14–16^. The latter, in turn, enhances the activity of the *VRN1* gene, thus inducing heading^14,16–18^. The *VRN2* gene is unaffected by low temperature as opposed to photoperiod. Short day inhibits while long day stimulates the expression of this gene^6,12,19,20^. In barley, both phenomic and gene expression analyses have confirmed that *VRN2* appears to be controlled by both the *CONSTANS* (*CO*) and *VRN1*, suggesting that this gene is a joint element in photoperiod and vernalization regulatory pathways^6,20^. *PPD1* (*PRR37*) is the major gene of photoperiod sensitivity^21–24^, and it acts with both the photoperiod regulating pathway and the circadian clock. In wheat, the dominant photoperiod insensitivity alleles in *PPD1* result in rapid heading irrespective of the photoperiod, an effect that is more pronounced under short photoperiods^25^. *VRN3* genes (*FT1*) are the central integrators of the vernalization and photoperiod pathways, and as such, these important genes are under the control of *VRN1*, *VRN2* and *PPD1* series^26–29^. The functional polymorphisms in these plant developmental genes have been thoroughly analysed, revealing the similarities and differences between the various cereal species^11,30–35^. In barley, it has already been described that different allele combinations of genes that are responsible for the regulation of vernalization requirement and photoperiod sensitivity lead to different plant development types^6,20^. However, the knowledge on the complex regulation system of plant development in cereals is still incomplete, necessitating further experiments.

One of the major differences between *Arabidopsis* and cereals appears in the relationship of floral differentiation and stem elongation. In *Arabidopsis*, these two processes normally occur in parallel; the first visible sign of the vegetative—generative turning point is the initiation of bolting from the vegetative rosette stage. Thus, the differentiation of the various floral organs takes place during the stem elongation. In the case of cereals, however, these two processes are separated in time. The vegetative—generative transition, followed by the floral differentiation and even the terminal spikelet formation in the case of wheat have all already taken place by the time the intensive stem elongation starts (the first visible sign for the latter is the appearance of the first node at the base of the stem). This phenomenon is well documented in the previous studies^36,37^, but it has not been considered in genetic models of cereal flowering regulation. In temperate cereals, photoperiod and low temperature vernalization are the two most decisive environmental factors determining the developmental processes of the plant^14,27^. In addition, several other factors fine-tune the heading or flowering, including the ambient temperature above vernalizing levels, the various characteristics of light, and water and nutrient availability that can show large variability within a short time frame, and it also varies by location and season.

The temperature above vernalizing levels has a more complex effect on plant developmental dynamics than the day length, as it may exhibit considerable deviation not only seasonally, but also at an annual or daily level^36,38–42^. Research on cereals proved that the ambient temperature influences not only the heading date, but also the number and development rate of leaves, tillers, and spikelets^43–45^. In addition to the light impulse, ambient temperature is also an important input signal in the entrainment of the circadian rhythm^46^. In barley seedlings germinating in the dark, the daily cycling in ambient temperature in itself is able to activate the circadian rhythm^47^. The ability to compensate for changes in external temperature is also characteristic of the circadian rhythm. The length of the period remains relatively constant in a wider range of ambient temperature, which is a fundamental element in keeping the cell metabolism at a relatively stable level, regardless of the external temperature.

Circadian rhythm with its incoming and outgoing regulation networks has been identified as the major component in continuously adjusting the various plant processes to the actual environmental conditions. As such, it must be also an important part of regulating the process of intensive stem elongation in cereals. The circadian rhythm is an internal regulatory mechanism (autonomous oscillator) by which plants synchronise their internal biological processes with daily changes in the various external environmental conditions including temperature and light^47–49^. Plant circadian clocks consist of multiple interconnected transcription / translation feedback loops^1^. The central oscillator mechanism of the clock is based on proteins providing feedback by directly or indirectly repressing their own activity or their own transcriptions from the corresponding genes. One of the central loops consists of three genes *TOC1*, and *CCA1*/*LHY*. Expression of genes encoding the transcription factors *CIRCADIAN CLOCK-ASSOCIATED 1* (*CCA1*) and *LATE-ELONGATED HYPOCOTYL* (*LHY*) peaks in the early morning^50,51^. In the morning, *CCA1* and *LHY* genes inhibit the expression of the *TIMING OF CAB EXPRESSION 1* (*TOC1*) gene, then in the late afternoon, the expression of the *TOC1* gene is increased, which reduces the expression of the *CCA1* and *LHY* genes (negative feedback loop)^52,53^. Subsequently, the expression of *PSEUDORESPONSE REGULATOR* (*PRR*) genes also increases until the evening hours (*PRR9*, *PRR7*, *PRR5*, *PRR3*, *PRR59*, *PRR73*, and *PRR95*)^51^. The *ARRHYTHMO* (*LUX*), *EARLY FLOWERING 3* (*ELF3*) and *ELF4* circadian genes show maximum expression in the evening and night periods, respectively and they are part of the evening complex (EC)^29,51^. In *Arabidopsis*, the circadian clock regulates the induction of *FT* and its critical transcriptional inducer *CONSTANS* (*CO*) via an external coincidence mechanism in which light coincides with an inductive window that is gated by the circadian clock. In this process, the clock component *GIGANTEA* (*GI*) plays an important role in forming a transcriptional complex via cycling changes in their protein abundance^1,54^. The study of the molecular bases of circadian clock in monocots has lagged behind^1^. Orthologs of circadian clock genes have been identified in cereals; however, their functions can largely differ from those described in *Arabidopsis*^55–59^. In barley and in wheat, to a smaller extent, several orthologs have been identified as the candidate genes of various *earliness *per se loci, which contributed significantly to the latitudinal spreading of these species^38,58,60–63^.

The photoreceptors are important input genes of the circadian oscillation, acting as the major sensors of light, but new results demonstrate the possible participation of some of its members in ambient temperature sensing as well^64,65^. In *Arabidopsis*, two major photoreceptor families have been identified, the five members of phytochromes (from *PHYA* to *PHYE*) and the cryptochromes (*CRY1* and *CRY2*)^57,66–69^. In cereals, the orthologues of *PHYA*, *PHYB*, *PHYC*, *CRY1* and *CRY2* have already been identified^57,70–72^. Their effects on flowering regulation have been proven mostly in association with photoperiodism and light spectral composition, but only limited information is available on their reaction to ambient temperature.

In summary, the regulatory mechanism of the plant circadian rhythm has been extensively explored in *Arabidopsis,* and several homologous genes have already been described in wheat, but few of these have been studied thoroughly. Much of this knowledge has been originated from diploid *Triticum monococcum* and tetraploid species with smaller genome sizes, or from specific crossing lines (RIL, NIL, mutant and transgenic lines). In addition, even less is known about the intrinsic relationship between circadian rhythm, major plant developmental genes, and photoreceptors in cultivated wheat varieties, and about the influence of secondary environmental elements such as ambient temperature. These environmental elements have a fundamental impact on the plant development. However, based on the results of our previous studies, we hypothesize that there may be significant differences in the molecular-genetic regulation of the intensive stem elongation phase between mono- and dicotyledonous plants.

Therefore, the aim of this research was to examine the possible associations between the major plant developmental, circadian, and photoreceptor genes in the diverse genetic backgrounds of three winter bread wheat cultivars as influenced by ambient temperature. For this purpose, the daily expression patterns of three vernalization response genes (*VRN1*, *VRN2* and *VRN3*), the major photoperiod sensitivity gene (*PPD1*), seven circadian clock genes (*CCA1*, *PRR95*, *TOC1*, *LUX*, *ELF3*, *GI* and *CO1*), and five photoreceptor genes (*PHYA*, *PHYB*, *PHYC*, *CRY1* and *CRY2*) were studied under controlled environmental conditions (phytotron). The application of long photoperiod (16 h) was common in all the environments, while the ambient temperature profile differed between the growth chambers. Two constant levels of temperature − 18 °C and 25 °C—were applied in the growth chambers. Vernalization, ambient temperature, daily timing, and genotype effects were all significant determinants of the variance in gene expressions, but their contributions varied remarkably with the different gene families. Unlike *Arabidopsis*, bread wheat has a very large and complex genome. Due to epistatic interactions between different allelic variants of plant developmental genes, it is often not possible to directly study the effect of a target gene. Consequently, here we can observe just indirect gene effect.

## Materials and methods

### Plant materials

Three hexaploid winter wheat cultivars (‘Mv Toborzó’ /AT1/, from Hungary, ‘Tommi’ /AT3/, from Germany and ‘Charger’ /AT20/, from United Kingdom) with different plant developmental and genetic diversities were included in the experiments. These plant materials originated from the GeneBank collection of the HUN-REN Centre for Agricultural Research, Hungary. They were selected on the basis of a previous study, where more details can be found on them^36^ (Fig. S1). In addition, the phenological responses of these genotypes to 18 °C and 25 °C have also been characterized in detail^36^. Based on the genotypic analysis with gene specific markers, all the three genotypes carry winter alleles of the major vernalization genes (*VRN-A1*, *VRN-B1* and *VRN-D1*) and in terms of photoperiod sensitivity, ʻMv Toborzóʼ carries the insensitive allele of *PPD-D1*, and the other two contain the sensitive allele type (‘Tommi’ and ‘Charger’), while all three cultivars carry the same sensitive allele in *PPD-B1*^36^ (Table S1).

### Experimental conditions and design

The controlled tests consisted of combinations of one photoperiod (16 h light period in a 24 h day) and two constant ambient temperature levels in three treatments (18 °C vernalized /18 °Cvern/, 18 °C unvernalized /18°Cuv/ and 25 °C vernalized /25 °C vern/), while all the other environmental factors were kept uniform across the treatments, in the phytotron (Phy) facilities of the Agricultural Institute, HUN-REN Centre for Agricultural Research, Hungary. The two applied temperatures represented optimal (18 °C) and supra‐optimal but no heat‐stress (25 °C) temperature levels. The same two Conviron PGV‐36 growth chambers (Controlled Environments Limited, Winnipeg, Canada) were used for these purposes, each representing one temperature level. The 16-h photoperiod was set in all environments at the same period of the day, from 5:00 AM (ZT0) to 21:00 PM (ZT16). The light source was metal halide lamps with a broad light spectrum (to create a spectrum which closely resembles natural light), and the light intensity was set to 240 μmol m^−2^ s^−1^ photosynthetic photon flux density—PPFD (suitable for the seedling stage), being the same as in the previous, corresponding study^36^. The humidity was constantly close to 70%. For all the experiments (18°Cvern, 18°Cuv and 25°Cvern), the plants were germinated in Jiffy® pots (Zwijndrecht, Netherlands) at room temperature for one week. This was followed (with the exception of unvernalized plants – 18°Cuv) by the vernalization treatment at 3 °C for 60 d under short photoperiod (9 h light period in a 24 h day) and low PPFD (20 μmol m^−2^ s^−1^). At the end of the vernalization the plants were at 1–2 leaf stages. They were then transplanted into pots 12 cm in diameter and 18 cm in height, with a soil capacity of 1.5 kg filled with a 4:1 mixture of garden soil and sand, and they were placed into the growth chambers. For the purpose of RNA sampling and apex examinations four plants per pots of each genotype were planted (50 pots holding 200 plants per genotype). The plants were left growing for two weeks, and then the collection of leaf samples began. After the completion of leaf tissues collections in each treatment, from the remaining seedlings the main tillers of three, two-week old plants per genotypes and treatments were dissected in order to examine the apex structure by measuring the length of the apex (APL) and evaluating the apex’ developmental phase using the Waddington scale^73^. For this purpose, the visible external leaves (ELN) and then the non-visible internal leaf sheaths (ILN) were removed by scalpel and counted, then the apex was photographed under a stereomicroscope (Alpha STO-PL6, Elektro-Optika Ltd, Érd, Hungary) equipped with length measuring capacity (ScopePhoto 3.0., ScopeTek Ltd, Bedford, UK). Furthermore, plant height (PH), and reproductive tillers (RT), were also measured. For determining the later developmental phases, two plants per pot and two pots per genotypes were also placed in each environment, in addition to the 50 pots of plants used for RNA sampling. The latter plants were raised till maturity or—in the case of the unvernalized treatment—until the termination of the experiment (at 120 days). This group of plants was not sampled for RNA isolation. In the latter sets of plants, two phenophases were defined based on the Zadoks’ scale^74^: appearance of the first main stem node (ZD31) and heading (ZD59), both expressed in days.

### Genetic characterisation of genotypes

Genomic DNA extraction was performed with the DNeasy® Plant Mini Kit (Qiagen Ltd, Hilden, Germany) from young leaves (100 mg) according to the manufacturer’s instructions. DNA samples were stored at -20 °C until use.

#### KASP marker system

KASP marker system was performed at the John Innes Centre (Norwich Research Park, Norwich, UK), according to the criteria provided by the company that developed the method. A detailed description of the method used and the reaction components and tools used can be found at https://www.lgcgenomics.com.

#### Determination of copy number variation of *VRN-A1*

The haploid copy number was determined using the multiplex TaqMan® assay method in collaboration with IDna Genetics Ltd (Norwich Research Park, Norwich, UK).

### Studying the gene‐expression levels

For studying the daily patterns of gene expressions of the major plant developmental, circadian and photoreceptor genes, collection of leaf tissues started two weeks after transplanting. In each treatment, the leaf sampling started at Zeitgeber time 1 (ZT 0 = 5:00 am, lights on), 1 h after the start of the light period; it was carried out every three hours, for two consecutive days (48 h), and it was finished with the ZT1 sampling of the 3rd day. Thus, at each time point, two data for each genotype and treatment became available from the two consecutive days, making it possible to include the day as a factor into the variance analyses. In each cases, the last fully expanded leaf was collected. One—one leaf of three plants from three separate pots were pooled for one biological replicate into a 1.5 ml Eppendorf tube that was immediately frozen in liquid N_2_, with a total of three biological replicates for each genotype (that is, a total of nine leaves per genotype, per sampling time). This approach was intended to minimize potential physiological differences between individual plants. Leaf sample was collected only once from each plant. For nighttime sampling, a low intensity green wavelength LED bulb (Philips-low energy 1W, Philips Electronics Ltd. Amsterdam, Netherlands) was used, which did not interfere with the normal function of the genes under studied. The leaf samples were stored in a – 80 °C freezer until further used.

Total RNA was isolated using the Qiagen RNeasy Plant Mini Kit (Qiagen Ltd, Hilden, Germany) after Trizol extraction, with an extra step of DNase treatment (with Qiagen Rnase-Free Dnase Set, Hilden, Germany—used to remove some remained gDNA contaminations) programmed in the QIAcube equipment (Qiagen Ltd, Hilden, Germany). The cDNA transcription was performed from 1.0 μg of total RNA using the RevertAid First Strand cDNA Synthesis Kit (Thermo Fisher Scientific Inc, Waltham, USA) with the standard protocol provided by the company. Changes in the expression levels were analysed for the vernalization response genes, *VRN1*, *VRN2* and *VRN3*, for the photoperiod sensitivity gene *PPD1*, for circadian genes, *CCA1*, *PRR95*, *TOC1*, *LUX*, *ELF3*, *GI* and *CO1*, and for photoreceptor genes *PHYA*, *PHYB*, *PHYC*, *CRY1* and *CRY2*, using the generic gene specific primers listed in Table S2. As the primers were designed by other research teams (Table S2), we checked the correctness of the primer sequences using a genome database (https://plants.ensembl.org). Furthermore, in all cases, the qRT-PCR melting curve profile and the length of the amplified region were found to be as expected. The primer pairs amplify all homoeoalleles (A, B and D copy) of each studied genes. The quantitative real‐time PCR was carried out in three biological and two technical replicates in a Rotor‐Gene Q equipment (Qiagen Ltd, Hilden, Germany) applying the SYBER‐Green technology of the company. There were 350 PCR reactions, with a total of 25,200 samples analysed with 72 samples per reaction. Gene expression was calculated using the Rotor-Gene software, which also considers the amplification efficiency. Relative concentration was normalized against the geometric mean of three housekeeping genes (Actin, β-tubulin and Ta30797), according to Vandesompele et al.^75^. The determination of the geometric average of three housekeeping genes and of the relative expression level of the gene of interests were carried out based on the following formulas:

$${\text{GAHKG}} = \sqrt[3]{{\left( {\Delta {\text{CtHKG}}1~ \times ~\Delta {\text{CtHKG}}2 \times \Delta {\text{CtHKG}}3} \right)}}$$

RQ = Ampl^GA_HKG_-ΔCt_TG_; GA, Geometric average; Ct, Cycle threshold; HKG: Housekeeping gene; RQ, Relative concentration; TG, Target gene; Ampl, amplification coefficient [corresponds to reaction efficiency of each sample (values of 2 = 100% efficiency)].

### Statistical analyses

The variance components were estimated by GenStat 18.0 (VSN International Ltd.) and SPSS 23.0 (IBM Data Science Community, Thomas J. Watson Research Center, Yorktown Heights, NY, USA) program packages using a restricted maximum likelihood method (REML). For the heatmap with the trees of hierarchical clustering, „gplots” and „dendextend” of the R program were used^76^. In the three different environments, the networks between all the examined genes were visualized with R qgraph 1.9.2^77^ on the 16 time-point data matrices of the three cultivars combined. The correlation heatmaps and the Principal Component Analysis (PCA) were visualized by metan 1.1.0 R package^78^ and by factoextra 1.0.7 R package (ggplot2)^79^. The chord diagrams were created using the circlize 0.4.16 R package^80^.

## Results

### Phenology in the context of temperature

Based on the population structure analysis^36^, the allelic composition of the *PPD-D1* gene impacted the phenotypic traits, including temperature response. On this basis, Kiss et al.^36^ distinguished three groups of cultivar response to temperature: photoperiod insensitive (PPD_ins), photoperiod sensitive (PPD_sens) and photoperiod sensitive with strong vernalization requirement (PPD_sens, VRN + +) (Fig. S1). The PPD_ins group was represented by AT1, while the PPD_sens, VRN +  + group by AT3 and AT20. Although the AT3 and AT20 varieties belong to the same temperature reaction group, their genetic distance is significant (Fig. S1). However, based on the gene-specific marker analysis, these selected varieties showed differences only in the copy number of *VRN-A1*, and in the allele types of *PPD-D1*, *RHT-B1* and *RHT-D1* among the main developmental genes that have been tested (Table S1). In the unvernalized treatment, none of the three varieties headed, and there was no significant difference in the developmental stage of their apices at two weeks of age. In vernalized treatments however, their development showed significant variation strongly influenced by temperature. AT1 (photoperiod insensitive) showed a significantly faster growth rate in 18°Cvern and 25°Cvern treatments compared to the two late cultivars (AT3 and AT20), illustrated by the average values of the parameters plant height (PH), apex length (APL), ZD31 and ZD59 (Fig. S2). There was no significant difference in the average heading of AT1 (ZD59) between 18 and 25 °Cvern treatments (Fig. S2). The two photoperiod sensitive cultivars (AT3 and AT20) suffered a significant delay in heading after the 25 °C treatment (106 and 118 days on average for replicates) compared to 18 °C, with the greatest delay in AT3.

### Daily expression patterns of the various gene families

Analyses of variance on the complete dataset of three genotypes × three environments × eight daily time points × two days revealed all four main factors being highly significant, but explaining a different portion of the variance that strongly depended on the gene families (Table S3). In general, the circadian genes were mostly influenced by the daily timing, the photoreceptor genes by the environment, and the plant developmental genes by the genotype and the environment. Within the gene families however, there were remarkable variations between the individual genes in the importance of the main factors. The 18°Cuv vs 18°Cvern and 18 °Cvern vs 25 °Cvern subsets were subjected to further analysis of variance to determine the most influencing environmental factor of each gene. These results are hereinafter demonstrated along the separate gene families. Figure S3 demonstrates the daily expression patterns of all the individual genes across the various genotypes and treatments. As the difference between the same time points of the two days was completely negligible compared to the main effects of genotype, environment and daily timing (Table S3), the 8 average values across a 24-h period are presented in these graphs, with the original data from the two days utilised in the determination of error bars and the significance levels (Fig. S3).

### Daily expression patterns of the major developmental genes

The expressions of all the three *VRN* genes were influenced to the largest extent both by the genotype and the environment (Table S3). Within this, *VRN1* and *VRN3* were more genotype dependent while the activity of *VRN2* was more influenced by the environment. Of the environmental factors, *VRN1* and *VRN3* were mostly determined by the vernalization treatment, but not by the ambient temperature. The activity of *VRN2* was, on the other hand, significantly modified both by vernalization and ambient temperature. In the case of *PPD1*, both environmental factors were the strongest compared to genotype. It is interesting to note that although all the four developmental genes showed daily fluctuations (Fig. S3), this factor explained more than 10% of the variance only in the context of uv – vern treatments for the *VRN* genes and in association with the genotype. For *PPD1*, the effect of daily timing, as a main factor, was above 10% in addition to its interaction with genotype.

**Fig. 1:**
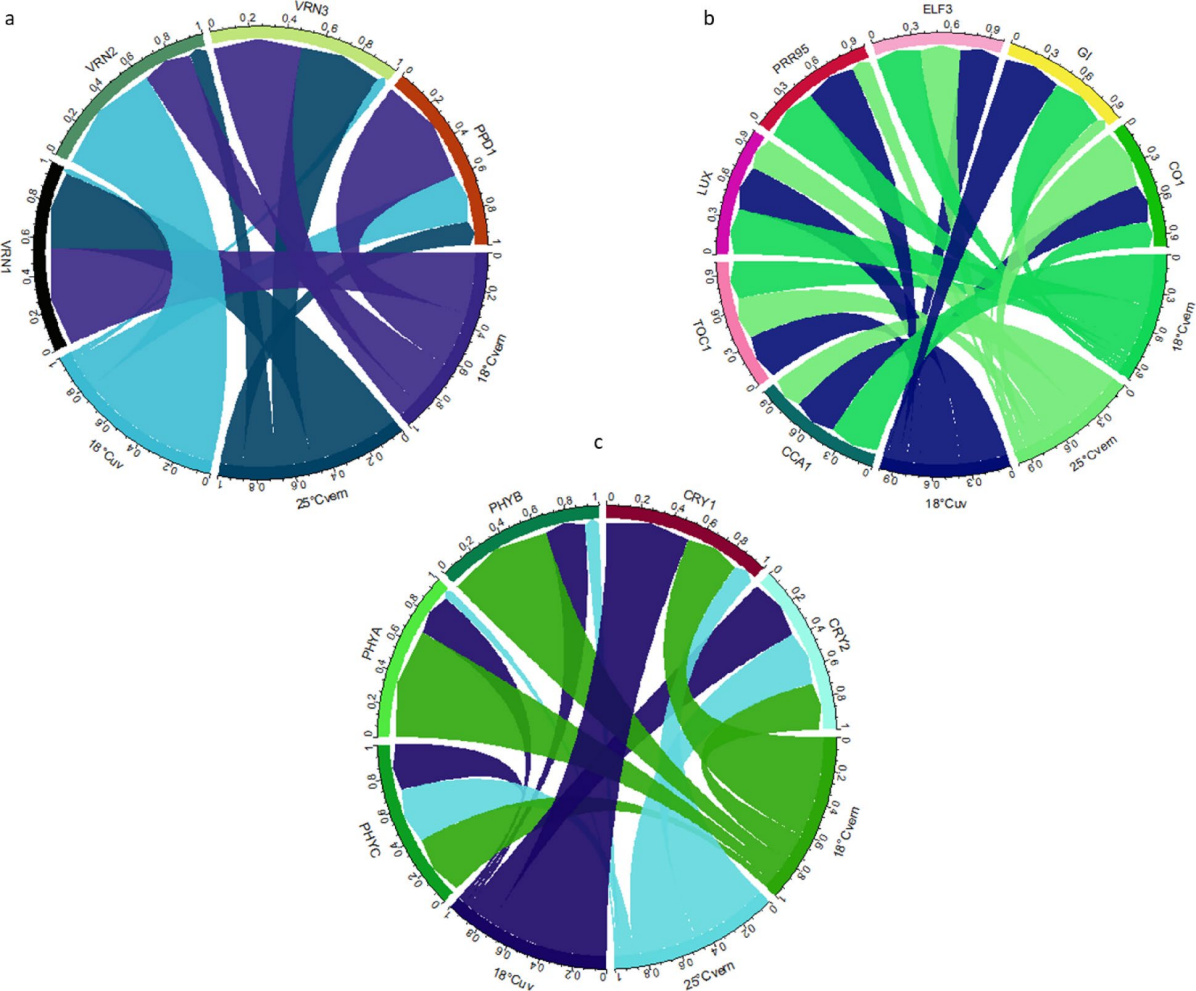
Chord diagram of the daily average expressions of the plant developmental genes (**a**), the circadian clock genes (**b**) and the photoreceptor genes (**c**) measured in the three treatments (25°Cvern – 25 °C vernalized, 18°Cvern – 18 °C vernalized and 18°Cuv – 18 °C unvernalized) based on the daily average gene activities of the three wheat cultivars. The ratio of values to each other expressed as a percentage. CCA1, CIRCADIAN CLOCK-ASSOCIATED 1; CO1, CONSTANS 1; CRY1, CRYPTOCHROME 1; CRY2, CRYPTOCHROME 2; ELF3, EARLY FLOWERING 3; GI, GIGANTEA; LUX, ARRHYTHMO; PHYA, PHYTOCHROME A; PHYB, PHYTOCHROME B; PHYC, PHYTOCHROME C; PPD1, PHOTOPERIOD1; PRR95, PSEUDORESPONSE REGULATOR 95; TOC1, TIMING OF CAB EXPRESSION1; VRN1, VERNALIZATION1 (APETALA1) ; VRN2, VERNALIZATION2; VRN3, VERNALIZATION3 (FLOWERING LOCUS T).

In unvernalized plants, the expression profile of the flowering enhancing gene, *VRN1,* was very low, and it did not show any characteristic daily rythm irrespective to the genotype (Table S4, Fig. S3a). After vernalization, a daily rhythm in *VRN1* gene expression could be detected, but this was highly specific both to the genotype and the environment. This finding is also well illustrated both by chord diagram and by heatmap of the daily average gene expression of the cultivars in the three treatments as compared to the percentage of average gene activities (Fig. 1a, Fig. S4a). The differences appeared not only in the daily timing of the peak expression, but also in its magnitude and the amplitude of the rhythm. In general, AT1, the photoperiod insensitive, early cultivar showed the highest peak expression followed by AT3, and then AT20, which showed a strong positive coincidence with the heading time (Fig. 1a, Fig. S3a, Fig. S4a).

It is interesting to note that only AT1 showed similar magnitude of expression in both vernalized environments with an environmental dependent specific daily rythm. In this cultivar, the peak expression of *VRN1* was at ZT4 in 18 °Cvern, which shifted to ZT13 in the 25 °Cvern (Table S4). In the case of the two late heading *PPD1* sensitive cultivars, the low *VRN1* expression in 25 °Cvern and only one peak at around ZT7 in the 18 °Cvern were the common features while they showed genotype specific differences in the daily rythm in both vern environments (Table S4, Fig. S3a).

Opposing to *VRN1* and *VRN3*, the activity of the flowering repressor gene, *VRN2,* was the highest in the unvernalized treatment with a distinct daily rythm that was quite similar for all the three cultivars. A significant decrease occurred after the early morning peak ZT1–4 that was followed by a second peak in the late afternoon ZT13–16 environments (Table S4, Fig. S3b). In the vernalized treatments, the *VRN2* activity and its daily pattern was more genotype specific. The level of decrease in the daily absolute values coincided with the earliness in all three vern treatments. Thus, they were the lowest in the early AT1 followed by AT3, and they remained the highest in AT20 where they were close to that measured in the unvernalized treatment (Fig. 1a, Fig. S3b, Fig. S4a). The daily fluctuation patterns were also genotype specific with the only exception of 25°Cvern. In the latter case, all three genotypes replicated at various extents the daily pattern detected in the unvernalized treatment.

In the case of *VRN3*, both the magnitude and the daily patterns were completely genotype specific during the various treatments. Only two tendencies were more general across the genotypes. The gene activity significantly increased due to vernalization, and the level of this increase coincided with the earliness of the genotype (Fig. 1a, Fig. S3c, Fig. S4a).

Environment impacted the expression levels of *PPD1* the most. In the unvernalized treatment, all three genotypes showed similar magnitude of the activity, and they were similar in the aspect that the daily fluctuation of this gene started with an early morning peak ZT1, but then the further courses were more genotype specific (Table S4, Fig. S3c). There was one aspect that showed connection with the allele type of *PPD-D1*, in the insensitive genotype: there was a night peak (detectable in all environments) that was missing from both sensitive genotypes (Fig. S3c). During the 18°Cvern, the *PPD1* activity was the highest for all three genotypes, and it was the only treatment where the magnitude of *PPD1* activity showed negative coincidence with the earliness (Fig. 1a, Fig. S3d, Fig. S4a). At 18°Cvern, the daily rhythm of the insensitive vs. sensitive genotypes could also be characterised with distinctly different patterns from each other (Fig. S3d). In the case of the insensitive AT1, the highest peak of early morning ZT1 was followed by a smaller peak in early afternoon (ZT13). On the other hand, three peaks of similar magnitude were characteristic to the daily oscillation of *PPD1* of the photoperiod sensitive genotypes: an early morning (ZT1), a midday (ZT7), and a late-night peak. These characteristics could not be detected during any of the remaining treatments.

### Daily expression patterns of the major circadian genes

The activity of the circadian genes both in the morning (*CCA1* and *PRR95*) and the evening (*TOC1*, *LUX* and *ELF3*) loops were mostly determined by the daily timing (Table S3). *CCA1* of the morning loop however, was the only one demonstrating the most stable daily cycling pattern irrespective to the genotypes and environmental conditions. The daily pattern accounted for 62.9–78.7% of its expressional variance in the different environments. It was slightly influenced by the genotype (σ^2^ = 15.2%***) in the context of unvernalized vs. vernalised treatments and by the temperature (17.6%***) in the context of 18 °C vs 25 °C (Table S3). The activity of *PRR95* was more influenced by the environment within which especially the strong effect of ambient temperature became evident in the vernalized treatments. For the genes in the evening loop, the effect of daily timing was more characteristic to *TOC1* and *LUX*. This was, however, modified to a smaller but significant extent by vernalization in the case of *TOC1* while by the ambient temperature for *LUX*. On the other hand, the environmental and genotypic plasticity of *ELF3* was its most characteristic feature with significant interactions detectable between all the factors in various combinations. The daily timing patterns of the two central integrator genes were much less characteristic. The activity of *CO1* was significantly influenced by genotype and environment (both vernalization and ambient temperature), while the activity of *GI* was more environment dependent. Of the environment factors, *GI* was mostly influenced by the ambient temperature but not by the vernalization treatment (Table S3).

The gene expression peaks of circadian genes with characteristic daily rhythms (*CCA1*, *PRR95*, *TOC1* and *LUX*) were basically specific, irrespective to the genotype and treatment. Based on our results, the *CCA1* gene expression peak was observed in the early morning hours. It was followed by *PRR95* around midday, then by the *TOC1* gene, together with *LUX* that were expressed in the late afternoon. The daily pattern of *ELF3* from the evening loop was not obvious, but its peak expression generally could be detected during the night hours.

Comparing the individual expression profiles of the three wheat genotypes under the different environments, significant differences could be observed for all the circadian genes, the extent of which was gene specific (Fig. S3e–k). There were significant variations even in the case of *CCA1* with the most conserved daily cycling pattern. However, it requires further studies to determine the phenotypic significance of these genotypic and environmental differences. This study highlights only the most interesting features including those that may show any possible associations with the allele phase of *PPD-D1* and thus earlier heading (Fig. 1b, Fig. S3e). This phenomenon was not quite characteristic to the morning loop genes but rather to the genes belonging to the evening loop. In the case of *TOC1*, all the three wheat genotypes showed the same shift in the daily peak due to the vernalization treatment (Fig. S3f.). In unvernalized plants, the *TOC1* expression peaked at ZT10, which shifted to the later hours ZT13–16 in the vernalized plants irrespective to the ambient temperature level. There was also an environmental dependent peak shift in the activity of *LUX*, but in this case, this shift did not quite distinguish the unvernalized treatment from the vernalized but rather the genotypes, and it coincided with the *PPD-D1* allelic phase (Fig. S3g). The peak shift in *LUX* activity of the *PPD-D1* insensitive genotype (AT1) was similar to that of *TOC1*. In the cases of the two *PPD-D1* sensitive genotypes (AT3 and AT20) however, *LUX* peaked in the unvernalized plants at the end of the light period, which moved earlier to ZT13 in 25 °C treatment and to ZT10 in the 18 °C treatment. In the case of *ELF3*, there were again remarkable differences between the insensitive and sensitive wheat cultivars (Fig. S3i). The amplitude of *ELF3* daily expression was narrow in the *PPD-D1* sensitive genotypes, with one or more smaller peaks depending on the environment. In contrast, a distinct night peak was characteristic to the insensitive cultivar AT1 in two of the three environments. The only exception was the 25 °C treatment where all the three genotypes similarly demonstrated the flattest expression curve of *ELF3*. It is also interesting to note that the three genotypes behaved similarly in the 18°Cuv treatment where in addition to the night peak, a second peak was apparent in the middle of daylight (ZT7–10).

The central circadian genes (*GI* and *CO1*) were characterised with mostly genotype and environment specific patterns. The expression profile of *GI* was more or less similar in the unvernalized plants of all the three cultivars with its maximum expression occurring at ZT13; there was only a slight difference between the genotypes in the maximum value (Fig. S3j). The behaviour of the cultivars differed most in the 18 °C vernalized treatment where the expression level in itself and compared to the non-vernalized level negatively coincided with the heading date. Vernalization decreased the activity of *GI* to the largest extent in AT1, the early heading cultivar. It was similar in magnitude but with a peak shift between the unvernalized and vernalized treatments for AT3, while it was the highest in the vernalized plants of AT20 with an accentuated peak at ZT13. This phenomenon however, was not apparent under the 25 °C treatments. As for *CO1*, its expression level was low in the unvernalized plants of the insensitive AT1 compared to the sensitive AT3 and AT20 (Fig. S3k). In addition, the largest difference between the genotypes became apparent under the 25 °C treatment. While in AT3, *CO1* showed similar activities in all the three vernalized treatments, AT1 was characterised with a significant night peak apparent only at 25 °C. However, it was AT20 that showed the most distinct and highest *CO1* activity, especially at 25 °C that was strongly elevated in late afternoon and after a short decrease before the end of the light period returned to that elevated level during the night.

### Daily expression patterns of the major photoreceptor genes

The expressions of all photoreceptor genes were influenced both by the genotype, environment, and daily timing (Table S3). Within this, *PHYA*, *PHYB* and *CRY1* were more environment dependent, while the activities of *PHYC* and *CRY2* were more influenced by the genotype, environment, and daily timing. In the case of *PHYA*, *PHYB* and *CRY1*, both environmental factors were the strongest compared to genotype. Of the environmental factors, *PHYC* and *CRY2* were mostly determined by the vernalization treatment but not by the ambient temperature. For these two genes, the effect of daily timing as a main factor was above 15%.

In the case of *PHYA* and *PHYB*, a significant high level of daily peak expression was shown in the 18°Cvern treatment (Table S4, Fig. S3l–m, Fig. S4c). This phenomenon was nearly tenfold higher than in 25°Cvern treatment (Fig. 1c, Fig. S3l–m, Fig. S4c). Furthermore, the appearance of daily peak expression of these genes was different in the three varieties. In the case of photoperiod insensitive variety (AT1), the peak expression was at 24:00 (ZT22) (Table S4, Fig. S3l–m). In the photoperiod sensitive varieties, two peaks were visible at ZT1 and at ZT10; for AT3 there were three peaks at ZT1 0, at ZT7–13, and at ZT22 for AT20 (Table S4, Fig. S4c). In vernalized treatments, *PHYC* showed a maximum activity at night in 25°Cvern for all three cultivars (Fig. S3n, Fig. S4c). A daily rythm in *CRY1* and *CRY2* genes expression was detected, but this was highly specific both to the genotype and the environment (Table S3). The differences appeared not only during the daily timing of the peak expression but also in its magnitude of the rhythm. The daily peak expression of *CRY1* was stronger in late heading varieties (AT3 and AT20) at 18 °C (regardless of the cold treatment) (Fig. S3o, Fig. S4c). Furthermore, a difference in the daily expression pattern was also observed between the three varieties. Under 25°Cvern condition, the transcriptional maximum of both genes was observed in the early morning (ZT1–4) and at night (ZT22) (Table S4, Fig. S3o).

### Interactions of flowering pathway genes in hexaploid wheat

**Fig. 2:**
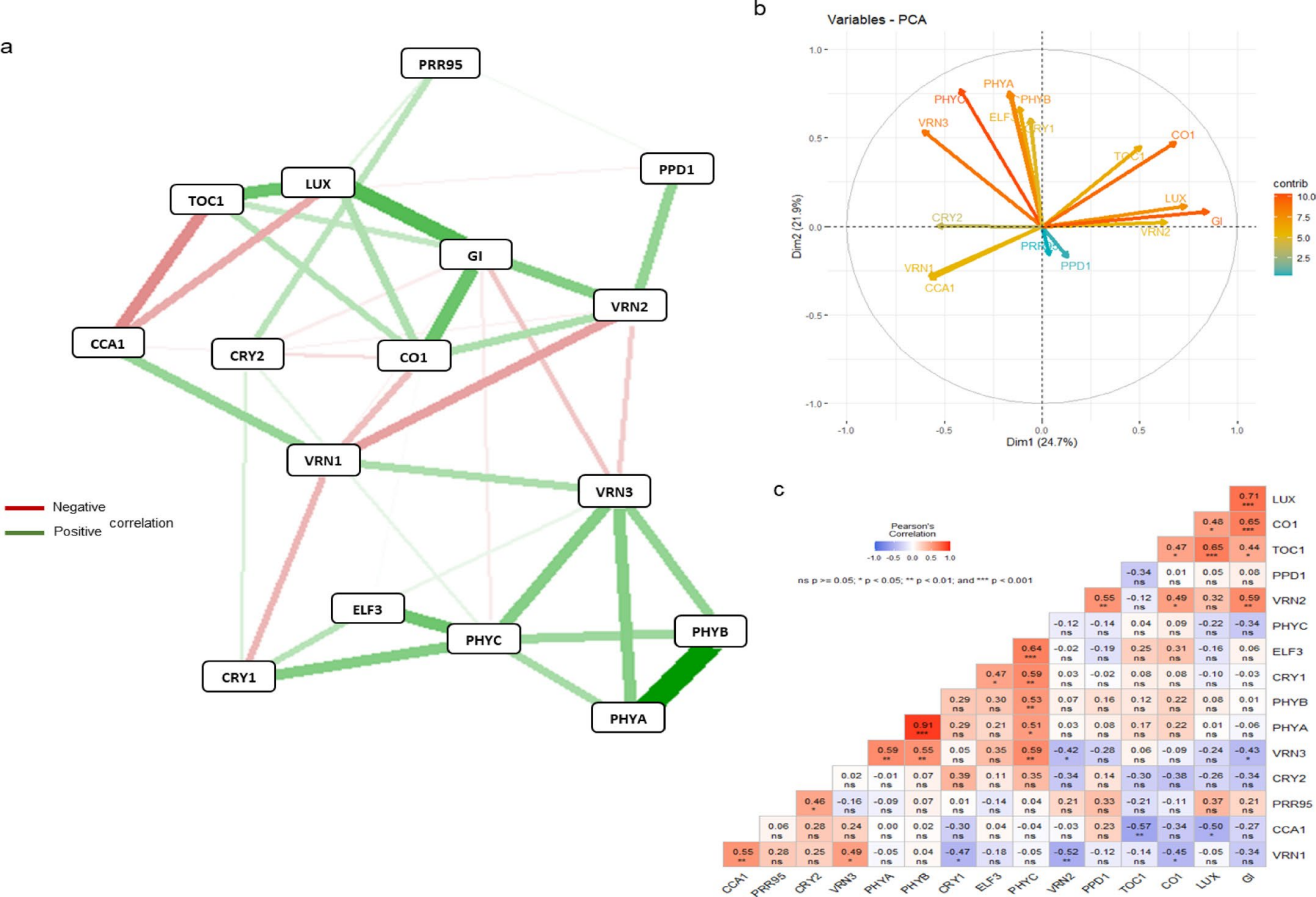
Gene network systems (**a**), PCA analysis (**b**) and correlation matrix (**c**) carried out on the data matrix of daily gene expression levels of 3 wheat cultivars in 18°Cvern (18 °C vernalized) treatment. The PCA analysis was presented as a supplementary, even more relevant illustration of the correlations between the genes investigated. CCA1, CIRCADIAN CLOCK-ASSOCIATED 1; CO1, CONSTANS 1; CRY1, CRYPTOCHROME 1; CRY2, CRYPTOCHROME 2; ELF3, EARLY FLOWERING 3; GI, GIGANTEA; LUX, ARRHYTHMO; PHYA, PHYTOCHROME A; PHYB, PHYTOCHROME B; PHYC, PHYTOCHROME C; PPD1, PHOTOPERIOD1; PRR95, PSEUDORESPONSE REGULATOR 95; TOC1, TIMING OF CAB EXPRESSION1; VRN1, VERNALIZATION1 (APETALA1) ; VRN2, VERNALIZATION2; VRN3, VERNALIZATION3 (FLOWERING LOCUS T).

**Fig. 3:**
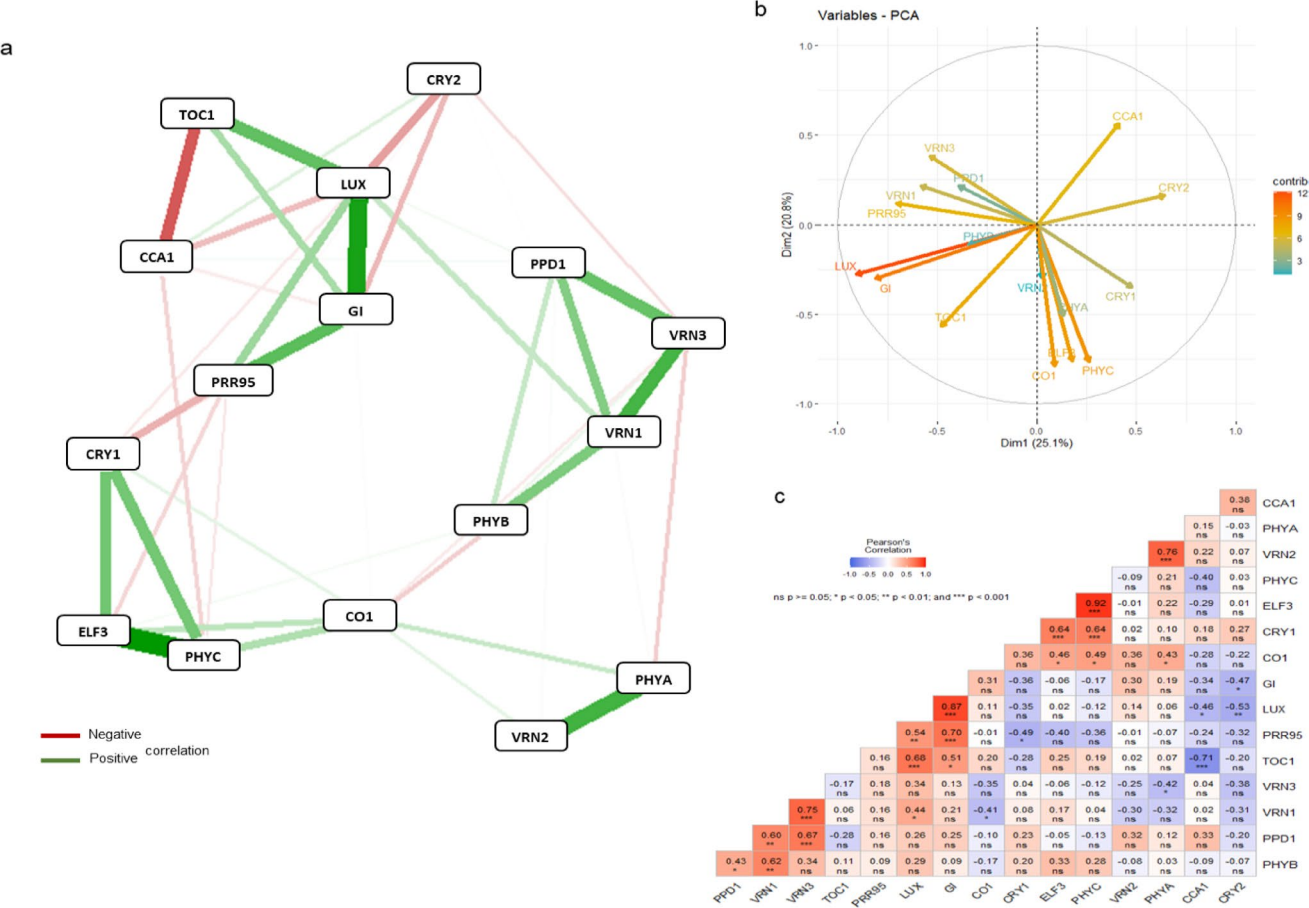
Gene network systems (**a**), PCA analysis (**b**) and correlation matrix (**c**) carried out on the data matrix of daily gene expression levels of 3 wheat cultivars in 25°Cvern (25 °C vernalized) treatment. The PCA analysis was presented as a supplementary, even more relevant illustration of the correlations between the genes investigated. CCA1, CIRCADIAN CLOCK-ASSOCIATED 1; CO1, CONSTANS 1; CRY1, CRYPTOCHROME 1; CRY2, CRYPTOCHROME 2; ELF3, EARLY FLOWERING 3; GI, GIGANTEA; LUX, ARRHYTHMO; PHYA, PHYTOCHROME A; PHYB, PHYTOCHROME B; PHYC, PHYTOCHROME C; PPD1, PHOTOPERIOD1; PRR95, PSEUDORESPONSE REGULATOR 95; TOC1, TIMING OF CAB EXPRESSION1; VRN1, VERNALIZATION1 (APETALA1) ; VRN2, VERNALIZATION2; VRN3, VERNALIZATION3 (FLOWERING LOCUS T).

**Fig. 4:**
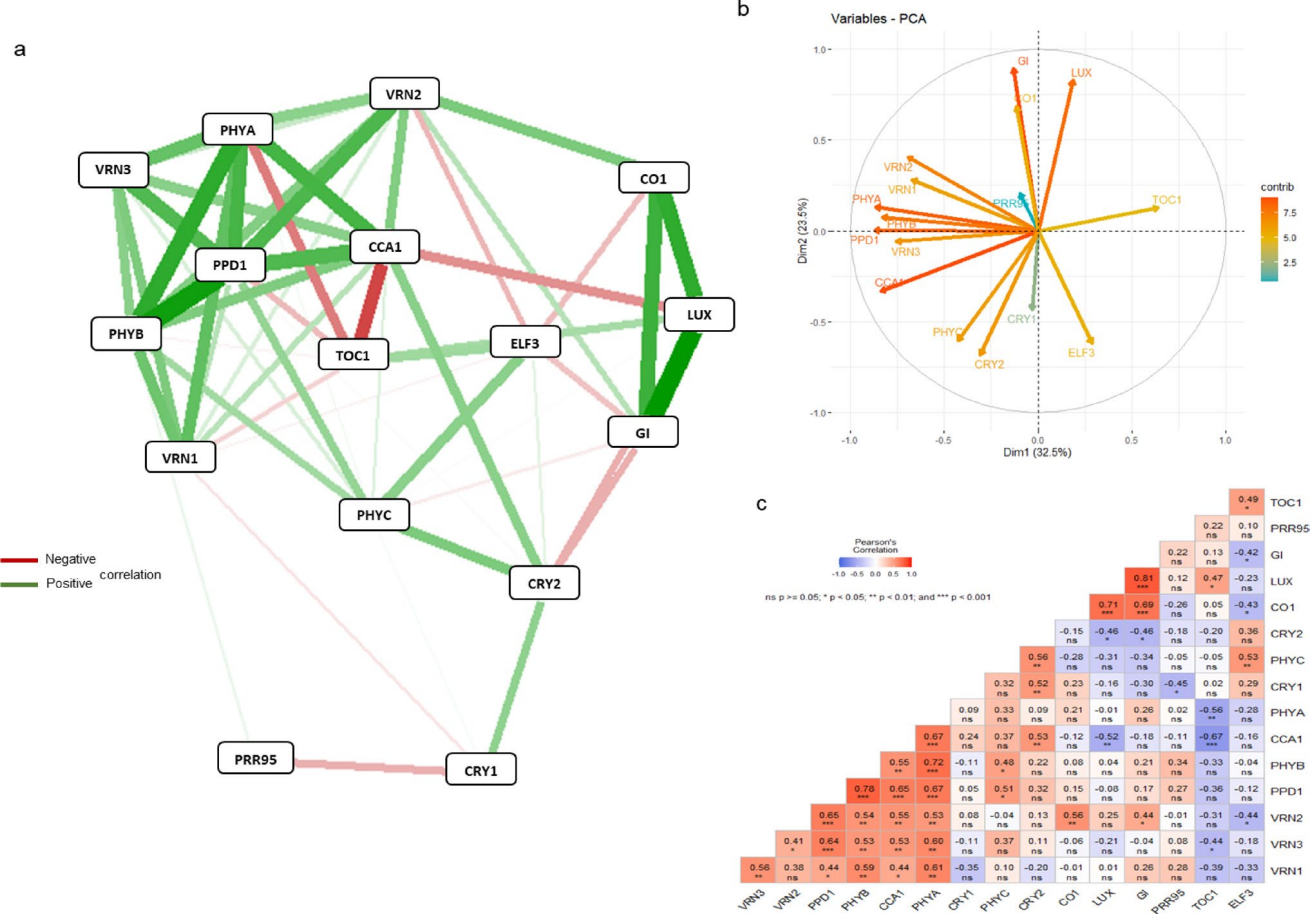
Gene network systems (**a**), PCA analysis (**b**) and correlation matrix (**c**) carried out on the data matrix of daily gene expression levels of 3 wheat cultivars in 18°Cuv (18 °C unvernalized) treatment. The PCA analysis was presented as a supplementary, even more relevant illustration of the correlations between the genes investigated. CCA1, CIRCADIAN CLOCK-ASSOCIATED 1; CO1, CONSTANS 1; CRY1, CRYPTOCHROME 1; CRY2, CRYPTOCHROME 2; ELF3, EARLY FLOWERING 3; GI, GIGANTEA; LUX, ARRHYTHMO; PHYA, PHYTOCHROME A; PHYB, PHYTOCHROME B; PHYC, PHYTOCHROME C; PPD1, PHOTOPERIOD1; PRR95, PSEUDORESPONSE REGULATOR 95; TOC1, TIMING OF CAB EXPRESSION1; VRN1, VERNALIZATION1 (APETALA1) ; VRN2, VERNALIZATION2; VRN3, VERNALIZATION3 (FLOWERING LOCUS T).

The network system of genes studied was significantly altered by the treatments. The fewest gene interactions were observed in 18°Cvern and 25°Cvern, while the most were visualized in 18°Cuv treatment (Figs. 2, 3, 4). In each treatment, the correlations between the expression levels of the 16 genes tested across the three wheat cultivars were also evaluated by principal component analysis (Figs. 2b, 3b, 4b).

The results confirmed the efficiency of the method used to visualize gene interaction (qgraph). The relationship between the three main *VRN* genes differed significantly between treatments, but the positive connection between *VRN1* and *VRN3* genes was maintained at a significant level, being the strongest in 25°Cvern treatment (Fig. 2). A strong positive association between *VRN1* and *CCA1* was observed in the 18°Cvern, but this phenomenon was not visualized in all other treatments. It is interesting to note that after vernalization, a negative association between *VRN2* and the other two *VRN* genes (*VRN1* and *VRN3*) was observed, which became more expressive at 18 °C. In this treatment, the positive association between *VRN2* and *PPD1* genes became stronger irrespective of vernalization, but this phenomenon was not observed in 25°Cvern treatment. A similar trend was found between *VRN2* vs *GI* and *VRN2* vs *CO1* genes. A strong positive correlation was found between *VRN3* and *GI* only in 18°Cvern treatment. The interaction between *PPD1* vs. *PHYB* and *PPD1* vs *CCA1* became more effective in 18°Cuv treatment. The interaction between *PPD1* and *VRN3* was expressed in most of the treatments with the only exception of 18°Cvern.

Three circadian genes (*LUX*, *TOC1* and *CCA1*) formed an internal network system in all treatments (Fig. 2, 3, 4). A positive correlation at a significant level detected between *TOC1* and *LUX*, while a negative relation at a significant level was observed to *TOC1* vs *CCA1* and to *CCA1* vs *LUX*. A strong positive correlation was observed between *GI* and *LUX* in all three environments. Furthermore, a strong positive correlation between the *GI* vs *LUX* vs *CO1* gene group was observed at 18 °C treatment, irrespective of vernalization. The interaction between *CO1* and *LUX* genes became more effective in 18°Cuv treatment. A positive correlation was described between *GI* and *CO1*, which became more significant at 18 °C, irrespective of vernalization treatment. A negative correlation was found between *ELF3* vs *GI*, *ELF3* vs *CO1* and *ELF3* vs *VRN2*. This finding was however only observed during 18°Cuv treatment. Based on our results, during 25°Cvern treatment, *CO1* showed a negative association with *VRN3*.

A positive correlation between one photoreceptor gene (*PHYC*) and one of EC (*ELF3*) was observed in all treatments with the strongest correlation in 25°Cvern treatment (Fig. 2, 3, 4). At 18 °C, the association between *PHYA* and *PHYB* genes became stronger irrespective of vernalization. Direct linkage of *PHYA* to *PHYB* and *PHYC* was abolished during 25°Cvern treatment. The relationships between the three phytochrome genes (*PHYA*, *PHYB* and *PHYC*) and *PPD1* were significantly altered by the treatments. While a strong positive association was observed between these genes during 18°Cuv, only *PHYB* in 25°Cvern and none of the phytochromes in 18°Cvern showed any correlation with the *PPD1* gene. A significant negative correlation was observed between *CRY2* vs *GI* and *CRY2* vs *LUX* in 25°Cvern and 18°Cuv treatment. A similar tendency was observed between *CRY1* and *PRR95*. A positive correlation is observed between *CRY1* and *PHYC*. This relationship was weakest during the 18°Cuv treatment.

## Discussion

The regulatory mechanism of the plant circadian rhythm has been extensively explored in *Arabidopsis,* and several homologous genes have already been described in wheat, but few of these have been studied thoroughly. Much of this knowledge has been originated from diploid *Triticum monococcum* and tetraploid species with smaller genome sizes, or from specific crossing lines (RIL, NIL, mutant and transgenic lines). In contrast to most studies, our aim was not to determine the effect of a single gene on ambient temperature using specific lines (RIL or NIL). The focus of this study was to describe the effect of different ambient temperature levels on the diurnal rhythm of expression of the main developmental (*VRN1*, *VRN2*, *VRN3* and *PPD1*), circadian (*CCA1*, *PRR95*, *TOC1*, *LUX*, *ELF3*, *GI* and *CO1*) and photoreceptor (*PHYA*, *PHYB*, *PHYC*, *CRY1* and *CRY2*) genes in juvenile plants and to investigate the interrelationships between them. For this purpose, three hexaploid wheat cultivars were included with different genetic backgrounds, based on the previous results^36^. However, we would like to emphasize, that the conclusions from the correlation matrix we presented do not imply necessarily the existence of functional relationships between the genes investigated. They give additional information on the interconnectedness of the genes in the complex genetic background of the hexaploid wheat genome and demonstrate how the various environmental factors – vernalization saturation and the ambient temperature in the current research – may influence this network. In the future, it will be essential to examine the homoeolog-specific regulation of the *VRN* and *PPD* genes using a broader range of varieties, and the studies should also be extended to description of specific effects occurring in later developmental stages.

**Fig. 5:**
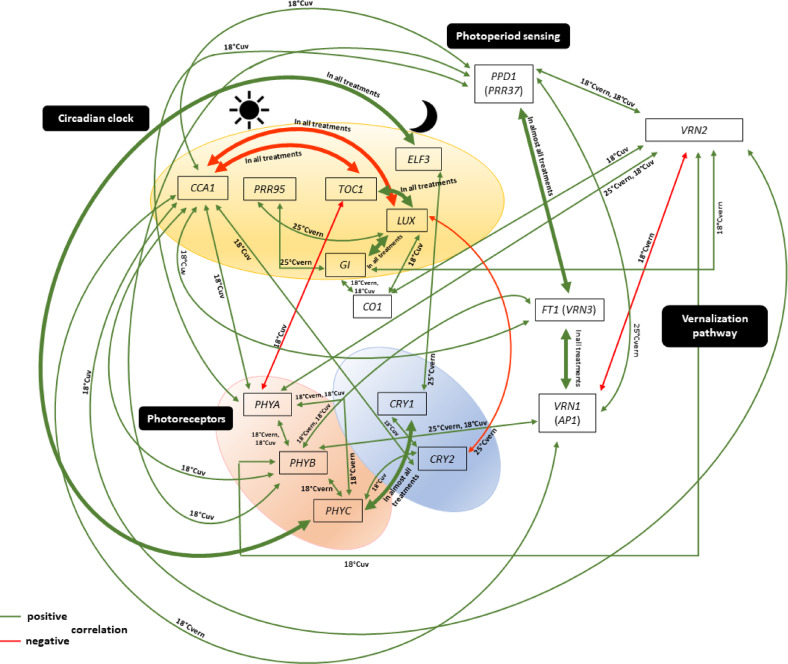
Summary of the major gene linkage systems (at least P ≤ 0.01) identified via comparing the daily gene expression patterns in three wheat cultivars. Boxes represent genes**.** Arrows indicate the positive (promotion—green) or negative (inhibition—red) associations between gene expression patterns. *Arabidopsis* orthologues of some key flowering genes in wheat are shown in parentheses. 18°Cvern/18 °C vernalized/, 18°Cuv/18 °C unvernalized/ and 25°Cvern/25 °C vernalized/. The presence of linkages between genes does not necessarily imply functional relationships among the investigated genes. CCA1, CIRCADIAN CLOCK-ASSOCIATED 1; CO1, CONSTANS 1; CRY1, CRYPTOCHROME 1; CRY2, CRYPTOCHROME 2; ELF3, EARLY FLOWERING 3; GI, GIGANTEA; LUX, ARRHYTHMO; PHYA, PHYTOCHROME A; PHYB, PHYTOCHROME B; PHYC, PHYTOCHROME C; PPD1, PHOTOPERIOD1; PRR95, PSEUDORESPONSE REGULATOR 95; TOC1, TIMING OF CAB EXPRESSION1; VRN1, VERNALIZATION1 (APETALA1) ; VRN2, VERNALIZATION2; VRN3, VERNALIZATION3 (FLOWERING LOCUS T).

The results identify that temperature significantly influenced the daily expression patterns of main developmental, circadian, and photoreceptor genes in hexaploid wheat. In the case of *VRN1* and *VRN3*, both the magnitude and the daily patterns were completely genotype and environment specific. The activity of these two genes significantly increased due to vernalization, and the level of this increased expression coincided with the earliness of the genotype. The positive relationship between *VRN1* and *VRN3* genes remained significant in all treatments (Fig. 5), which confirms the finding that the close relationship between these two genes is essential for the regulation of flowering^17,81,82^.

It has been demonstrated that during vernalization, cold gradually activates the *VRN1* gene, which also stimulates the *VRN3*. This gene feeds back to the *VRN1* to further increase its activity, thus inducing heading. Based on our results, the expression of *VRN1* and *VRN3* in the early cultivar (AT1) was 3–tenfold higher than in the late cultivars (AT3 and AT20), independent of temperature treatment, which showed a strong positive coincidence with the heading time (Fig. S2). The vernalized *VRN2* gene showed an explicit diurnal activity in late heading cultivars. This effect became most significant at 18 °C and 25 °C for the AT20 causing late heading similarly to the unvernalized treatment. Furthermore, a strong positive association between *VRN2* and *CO1* was observed at 18 °C, irrespective vernalization (Fig. 5). Mulki and von Korff^83^ described that *HvVRN2* was up-regulated by *HvCO* in barley. It can be assumed that *VRN2* is an integrator between light and vernalization pathway in temperate cereals^51^, and this gene may still play an important role in the regulation of plant development even after vernalization extending its function already reported by Yan et al.^4^, Fjellheim et al.^84^ and Kiss et al.^36^. The diurnal rhythm of *PPD1* gene was also significantly affected by genotype and temperature, which can be the consequence of using generic primers amplifying *PPD-A1*, *PPD-B1* and *PPD-D1* at the same time. These three genes participate directly in determining photoperiod sensitivity and they differ not only in the causal polymorphisms but also in the daily cycling pattern. The gene expression level of the photoperiod sensitive allele was defined as very low in the early morning, peaking in the morning, and declining again^22,85,86^. In contrast, photoperiod insensitive alleles do not show this diurnal fluctuation, and the gene activity level is consistently elevated^11,22,26,29,85,86^. The results of this study also support these findings in most treatments with the only exception of 18°Cvern. Here, the *PPD1* activity was the highest for all three genotypes, and it was the only treatment where the magnitude of *PPD1* activity showed negative coincidence with the earliness. This study also found a significant change in the strength and number of *PPD1* vs. circadian gene interactions. This phenomenon may correspond to the finding that the *PPD1* gene is closely related to the *Arabidopsis PRR7* gene. This gene also plays a role in the perception of light and temperature in the circadian rhythm, so the *PPD1* gene may have a similar role in cereals^36,87^. Furthermore, a strong positive correlation between *PPD1* and *VRN3* gene was observed in almost all treatments (excluding 18°Cvern) (Fig. 5), supporting previous studies^26,29^. Therefore, the wheat *VRN3* gene can be a central gene in the vernalization and photoperiod regulatory pathways that integrates different environmental signals^28^.

The activity of both morning (*CCA1* and *PRR95*) and afternoon (*TOC1*, *LUX* and *ELF3*) loop circadian genes were mostly determined by daily timing, irrespective of vernalization and temperature treatments. The central genes involved in daily circadian rhythm regulation, *CO1* were significantly influenced by genotype, while the activity of *GI* was more environment dependent. Based on the previous experiments^50,51,88–94^ with *Arabidopsis*, the expression peak of *CCA1* and *PRR95* genes are observed in the early morning hours, followed by *TOC1* around in late afternoon. Finally, the transcription peak of EC genes (*LUX*, *ELF3* and *ELF4*) was detected at night. In the morning, the *CCA1* and *LHY* genes inhibit the expression of *TOC1* and EC genes respectively, then in the late afternoon, the *TOC1* repress the transcription of the *CCA1* and *LHY* genes (negative feedback loop) and also impacts its own function, reducing its own activity. The *LUX*, *ELF3* and *ELF4* genes inhibit the *PRR5*, *PRR7*, *PRR9*, *TOC1* and *GI* genes, resulting in a decrease in the inhibition of *CCA1* and *LHY* genes, thus initiating a new diurnal cycle. This study identified similar functions: *CCA1* expression being the highest at early morning, while *TOC1* expression at evening, and a significant negative association between them irrespective to the environment. The homologs of *CCA1*/*LHY* in temperate grasses are responsive to photoperiod and temperature regulation, but their effects on flowering time remain unknown^47,60^. This study found that, indeed, the level of expression of *CCA1* was significantly influenced by the temperature and genotype. For AT1 (early heading type), it was 18 ºC highest throughout of the day, while for both AT3 and AT20 (late heading types) the morning peak was the highest at 25 ºC. The *TOC1* gene was also identified in wheat, which has been characterized to have an evening expression peak^95^, matching the diurnal functional pattern of its *Arabidopsis* homologue gene^96^. However, the transcriptional mechanism and gene interaction of this gene are not yet known^97^. The *PRR95* expression being the highest at the middle of the day while *LUX* and *ELF3* expression at evening and night. This study found a significant negative association between *CCA1* and *LUX* while a significant positive correlation between *TOC1* and *LUX*, irrespective to the environment (Fig. 5). The close, temperature-independent relationship between these major circadian genes may also illustrate their fundamental role in the floral regulatory system even for varieties with different genetic backgrounds. In *Arabidopsis* (long day plant), the circadian clock regulates the induction of *FT* and its critical transcriptional inducer *CO* via an external coincidence mechanism in which light coincides with an inductive window that is gated by the circadian clock^1^. In this process, the clock component *GI* has an important role in forming a transcriptional complex with another factor (*FLAVIN-BINDING KELCH REPEAT F-BOX 1 /FKF1*/) via cycling changes in their protein abundance. In long days, the phase of peak *GI* accumulation coincides with that of *FKF1* before dusk; thus, their complex is able to degrade the *CYCLING DOF FACTOR 1* (*CDF1*) repressor of *CO* in late afternoon relieving the transcriptional repression of *CO*, leading to the accumulation of stable CO protein, which is able to activate *FT*. The positive effect of *HvCO1* on *HvFT1* expression has been described in barley^98^, but little information is available in wheat. In accordance with these schemes, this study did not find strong positive relationship between the *CO1* and *VRN3* (*TaFT*) genes. Furthermore, in the long-day experiment, *GI* expression peaked during the late afternoon in most of the cases mostly irrespective to the environments. At 18 °C (including 18°Cuv), the activity of this gene increased 3–fivefold compared to the 25 °C experiment, irrespective of genotype. A similar tendency was observed for the *PRR95*. In contrast, in barley, Ford et al.^47^ described, that increased expression of these genes was induced by increasing temperature (from 15 to 25 °C). The activation peak of *CO1* however, significantly varied with the genotypes and treatments. In addition, a direct association between these two genes was only found in 18°Cuv and 18°Cvern treatments, but not in 25 °C environment (Fig. 5). Zhao et al.^99^ also described the positive correlation between the wheat *GI* and *CO* genes. Furthermore, a strong positive correlation was observed between *GI* and *LUX* gene irrespective of the environment (Fig. 5). A negative interaction between wheat *ELF3* and *GI*, consistent with the function of the *Arabidopsis* homolog gene has been described^55,58^. Based on the results, this phenomenon was only confirmed in 18°Cuv treatment (Fig. 5). Furthermore, a similar tendency was also observed between *ELF3* vs *CO1* and *ELF3* vs *VRN2*, which was eliminated by the cold treatment.

*PHYA*, *PHYB* and *CRY1* genes were strongly affected by the environment, while *PHYC* and *CRY2* genes were significantly influenced mainly by genotype, environment, and daily timing. *PHYA* and *PHYB* genes showed a nearly tenfold increase in average daily gene activity in 18°Cvern compared to 25°Cvern treatment. It has been described that the *PHYB* photoreceptor in *Arabidopsis* also functions as a temperature transmitter. However, this has not yet been tested in temperate cereals^64,65^. Based on the correlation data matrices, in bread wheat, the *PHYA* may play a similar role to *PHYB*. Furthermore, a strong positive correlation of *PHYA* was identified with *VRN3* (in 18°Cvern and 18°Cuv) and *VRN2* (in 25°Cvern and 18°Cuv) (Fig. 5). In their work on mutant tetraploid wheat, Alvarez et al.^100^ described the important role of light in the interaction of *ELF3*, *PHYB* and *PHYC* having a direct effect on *PPD1* gene. Woods et al.^107^ demonstrated, that *ELF3* express downstream from *PHYC* and acts as a repressor of *PPD1* in the photoperiod flowering pathway of *Brachypodium distachyon*. This study demonstrates a significant level of positive correlation between *PHYC* and *ELF3* that was observed in all treatments (Fig. 5). A strong positive association was also observed between the three phytochrome genes (*PHYA*, *PHYB* and *PHYC*) and *PPD1* in 18°Cuv treatment (Fig. 5). In wheat, a positive co-expression role of *PHYC* and *PHYB* in flowering has already been described, but no information was available on the *PHYA*^57,70,72^. The results of this study highlighted the fact that not only *PHYB* and *PHYC* showed a strong association with plant development genes but also *PHYA* (Fig. 5), probably in a redundant manner. A critical element of the photoperiod pathway is the light-mediated stabilization of the CO protein, in which *PHYA*, *CRY1* and *CRY2* and also *PRR*s play important roles in *Arabidopsis* when abundantly present^101^. In this experiment, positive association between *CO* and *CRY1* was detected in 25°Cvern only; between *CO* and *PHYA* in 25°Cvern, but no other connections reached the significance level. The relationship between the *VRN1* vs *VRN3*, *CCA1* vs *TOC1* vs *LUX* vs *GI* and *PHYC* vs *ELF3* genes play a fundamental role in the vegetative-generative transition of apex showing a strong correlation at a significant level irrespective of ambient temperature and vernalization treatment (Fig. 5).

This study highlights the complex relationships between gene families determining early development in hexaploid wheat cultivars, which were significantly influenced by different ambient temperatures. The genetic plasticity present in varieties in response to different environmental cues can be an important factor in the ecological adaptability. Therefore, a better understanding of the mechanisms of action of environmental factors can help to produce new varieties that can adapt to future changing environmental conditions.

## Conclusions

The molecular genetic regulation of the transition between the vegetative and generative stages in the model plant *Arabidopsis* has been most extensively studied. In the contrast, the molecular genetic processes of plant development in wheat are much less well understood, and only the major components of the vernalization and photoperiod regulation pathways have been identified in detail. Studying the genes that determine flowering in *Arabidopsis* may also provide a good basis for investigating orthologous genes in wheat. In hexaploid wheat varieties with different developmental patterns we found a positive relationship between *VRN1* and *VRN3* genes irrespective of ambient temperature treatments. The vernalized *VRN2* gene showed an explicit diurnal activity in late heading cultivars and this gene may still play an important role in the regulation of plant development after cold treatment. The diurnal rhythm of *PPD1* gene was significantly affected by temperature. At 18 °C, the vernalized *PPD1* activity showed negative coincidence with the earliness. The close temperature-independent relationship between the investigated major circadian genes (*CCA1*, *PRR95*, *TOC1*, *LUX*, *ELF3*, *GI* and *CO1*) may also illustrate their fundamental role in the floral regulatory system. *PHYA*, *PHYB* and *CRY1* genes were strongly affected by environment, while *PHYC* and *CRY2* genes were significantly influenced mainly by genotype, environment and daily timing. This study is one of the few works which investigate the diurnal expression patterns of key developmental, circadian, and photoreceptor genes in the diverse genetic background of bread wheat cultivars and under different ambient temperatures. This might open the floor for further research in this field which, in turn, could provide more detailed insights into the genetic regulation of cereal plant development across a broad genetic background.

## Electronic supplementary material

Below is the link to the electronic supplementary material.

Supplementary Material 1

## Data Availability

The data supporting the findings of this study are available within the manuscript or supplementary information files, as well as from the corresponding authors upon reasonable request.
