# Supplementary material for: Ambient temperature influenced co-expression network of major developmental, circadian, and photoreceptor genes in bread wheat
Source: Sci Rep. 2025 Jul 30;15:27751. doi: 10.1038/s41598-025-12424-6 (PMC12311187; doi:10.1038/s41598-025-12424-6)
Supplement: Supplementary file 1 — Supplementary Material 1 [file 41598_2025_12424_MOESM1_ESM.docx]

**SUPPORTING INFORMATION**

**Ambient temperature influenced co-expression network of major developmental, circadian, and photoreceptor genes in bread wheat**

**Tibor Kiss^1,2*^, Ádam D. Horváth^1^, András Cseh^1^, Zita Berki^1^, Krisztina Balla^1^, Marianna Mayer^1^, Viola Tóth^1^, Ildikó Karsai^1*^**

^1^ HUN-REN Centre for Agricultural Research, Agricultural Institute, H-2462 Martonvásár, Hungary

^2^ Food and Wine Research Institute, Eszterházy Károly Catholic University, H-3300 Eger, Hungary

*corresponding authors: [kiss.tibor@atk.hu](mailto:kiss.tibor@atk.hu), [karsai.ildiko@atk.hu](mailto:karsai.ildiko@atk.hu)

**
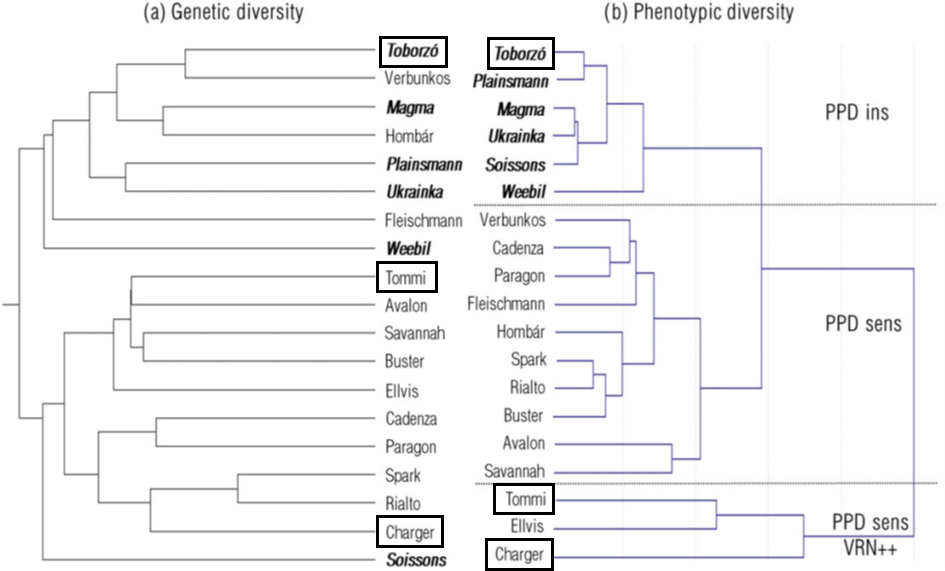
**

**Figure S1.** Genetic and phenotypic diversities between the 19 wheat cultivars, based on (a) 4971 SNP markers and (b) 16 phenology traits measured at six various environments combining two photoperiod × three temperature regimes (Wheat genotypes with bold italics carry the *PPD‐D1* insensitive allele) based on Kiss et al.^36^.

Abbreviations: (PPD_ins) – photoperiod insensitive allele type, (PPD_sens) – photoperiod sensitive allele type, (VRN++) – strong vernalization requirement

**Table S1.** Allele distribution of the main developmental genes in the three wheat cultivars examined.

Abbreviations: *ELF3* (*TaELF3-1D*) – *EARLY FLOWERING 3*, *PPD1 (PPD-D1, PPD-B1)* – *PHOTOPERIOD1*, *VRN1* (*VRN-A1*, *VRN-B1*, *VRN-D1*) – *VERNALIZATION1* (*APETALA1*), *RHT1* (*RHT-B1*, *RHT-D1*) – *REDUCED HEIGHT 1*

| **Abbr** |  | **AT1** | **AT3** | **AT20** |
| --- | --- | --- | --- | --- |
| **Name** |  | ʻMv Toborzóʼ | ʻTommiʼ | ʻChargerʼ |
| **Origin** |  | Hungary | Germany | Great Britain |
| ***VRN-A1a* (**Intron 1 deletion**)** |  | winter | winter | winter |
| ***VRN-A1b* (**Promoter deletion**)** |  | winter | winter | winter |
| ***VRN-A1* (**Exon 4 SNP**)** |  | long vernalization | long vernalization | long vernalization |
| ***VRN-A1* (**Haploid copy number**)** |  | 2 | 2 | 3 |
| ***VRN-B1a* (**Intron 1 deletion**)** |  | winter | winter | winter |
| ***VRN-D1a* (**Intron 1 deletion**)** |  | winter | winter | winter |
| ***PPD-D1a* (**Promoter deletion**)** |  | photoperiod insensitive | photoperiod sensitive | photoperiod sensitive |
| ***PPD-B1a* (**Exon 3 SNP**)** |  | photoperiod sensitive | photoperiod sensitive | photoperiod sensitive |
| ***TaELF3*-1D** |  | late heading | late heading | late heading |
| ***RHT-B1*** |  | dwarf | wild | wild |
| ***RHT-D1*** |  | wild | dwarf | dwarf |

**Table S2.** Details of the generic primers used for qRT-PCR analysis. Primer specificity was verified by in silico validation against the genome database (https://plants.ensembl.org), confirming successful amplification of the target gene regions.

Abbreviations: *CCA1* – *CIRCADIAN CLOCK-ASSOCIATED 1*, *CO1* – *CONSTANS 1*, *CRY1* – *CRYPTOCHROME 1*, *CRY2* – *CRYPTOCHROME 2*, *ELF3* – *EARLY FLOWERING 3*, *GI* – *GIGANTEA*, *LUX* – *ARRHYTHMO*, *PHYA* – *PHYTOCHROME A*, *PHYB* – *PHYTOCHROME B*, *PHYC* – *PHYTOCHROME C*, *PPD1* – *PHOTOPERIOD1*, *PRR95* – *PSEUDORESPONSE REGULATOR 95*, *TOC1* – *TIMING OF CAB EXPRESSION1*, *VRN1* – *VERNALIZATION1* (*APETALA1*), *VRN2* – *VERNALIZATION2*, *VRN3* – *VERNALIZATION3* (*FLOWERING LOCUS T*)

| **Primer name** | **Gene** | **Sequence (5′ – 3′)** | **Associated wheat sequences (Genes)** | **Reference** |
| --- | --- | --- | --- | --- |
| q-VRN1-F1  q-VRN1-R1 | *VRN1* | GAACAAGATCAACCGGCAGGTGAC  GGAGAAGATGATGAGGCCGACCTC | TraesCS5A02G391700  TraesCS5B02G396600  TraesCS5D02G401500 | Boussicut, R., Taulemesse, F. and Allard, V.  (INRA, UMR 1095 GDEC, France) |
| q-ZCCT2-F1  q-ZCCT2-R1 | *VRN2* | CATCGTGCCATTCTGCGGG  CCCTGTACCTCATCACCTTCGCCT | TraesCS5A02G541200  TraesCS5B02G180400  TraesCS5D02G361200 |  |
| q-VRN3-F1  q-VRN3-R1 | *VRN3* | TCAGGGTGACCTTCGGGAACAG  TCATCTCATTGCCGCCCACC | TraesCS7A02G115400  TraesCS7B02G013100  TraesCS7D02G111600 |  |
| F1  R1 | *PPD1* | GTCGTCACCGCCCTGCTCC  TCGTGGTTCATGATCCTGCCG | TraesCS2A02G081900  TraesCS2B02G344100  TraesCS2D02G079600 |  |
| TOC1-F  TOC1-R | *TOC1* | GAGGATAGCTGAGTCATCTGC  GTCGGTCGCACCAGAGAAAT | TraesCS6A02G227900  TraesCS6B02G253900  TraesCS6D02G207100 | 102 |
| CCA1-F  CCA1-R | *CCA1* | CAAGGTCTTCTCCCTCTTTTTGCTC  GTTGACCTTGCTCCTGAGCTACTTG | TraesCS7A02G299400  TraesCS7B02G188000  TraesCS7D02G295400 | 103 |
| LUX-F  LUX-R | *LUX* | ACAAGCGGTTCGTGGAGGTG  GACGTAGAGGCGGTACTTCTGGAG | TraesCS3A02G526600  TraesCS3B02G594300  TraesCS3D02G531900 |  |
| PRR95-F  PRR95-R | *PRR95* | GTCTGGGTTCCTCCTACTCTCCAC  GCATTTGAAAACCATGCTAACTGC | TraesCS5A02G320300  TraesCS5B02G320500  TraesCS5D02G326200 |  |
| TaELF3-F  TaELF3-R | *ELF3* | GTGGGATCGACAGACCTC  CGACGCGTTCCTTCC | TraesCS1A02G443200  TraesCS1B02G477400  TraesCS1D02G451200 | 58 |
| GI-07-F  GI-07-R | *GI* | caattgccacaccaagtgcta  tgatgaattcagaggtaacaaacca | TraesCS3A02G116300  TraesCS3B02G135400  TraesCS3D02G118200 | 22 |
| CO1-07-F  CO1-07-R | *CO1* | ccataatcgcagtggcaac  tggcttctctctccttggagc | TraesCS7A02G211300  TraesCS7B02G118300  TraesCS7D02G213000 |  |
| CRY1a-17-F  CRY1a-17-R | *CRY1* | CAGCCTCAGACTCGGATGG  CTGAAGTGGATGGTGCTATGCC | TraesCS6A02G203300  TraesCS6B02G224100  TraesCS6D02G187200 | 104 |
| CRY2-17-F  CRY2-17-R | *CRY2* | CTGGTGGTTATTTAGTTGGGTCGT  GTACCACTATCGGCACTGTCATC | TraesCS6A02G223700  TraesCS6B02G257600  TraesCS6D02G211700 |  |
| PHYA-17-F  PHYA-17-R | *PHYA* | TGCAGCACATTCAGAGAGGG  CCCTAGTGCCTTGTGCAGAG | TraesCS4A02G262900  TraesCS4B02G052000  TraesCS4D02G052200 |  |
| PHYB-17-F  PHYB-17-R | *PHYB* | GACCTGTCGCCTCACCACT  CAGCGAGATCTCCCGTGC | TraesCS4A02G122500  TraesCS4B02G182400  TraesCS4D02G183400 |  |
| PHYC-rtF  PHYC-rtR | *PHYC* | ACTACCCGGCAACTGACATC  GAGCCACAGAGGCTGATAGG | TraesCS5A02G391300  TraesCS5B02G396200  TraesCS5D02G401000 | 63 |
| Ta30797-F  Ta30797-R | *PGD* (Phosphogluconate dehydrogenase) | GCCGTGTCCATGCCAGTG  TTAGCCTGAACCACCTGTGC | TraesCS6A02G148400  TraesCS6B02G176600  TraesCS6D02G137700 | 105 |
| β-tubulin-F  β-tubulin-R | *Tubulin* | CCATCAGTTGGTTGAGAATGC  CAAAGCTGGGAGTGGTCA | TraesCS1A02G309700  TraesCS1B02G320800  TraesCS1D02G309200 | Boussicut, R., Taulemesse, F. and Allard, V.  (INRA, UMR 1095 GDEC, France) |
| Act-Shim09-F  Act-Shim09-R | *Actin* | TATGCCAGCGGTCGAACAAC  GGAACAGCACCTCAGGGCAC | TraesCS1A02G274400  TraesCS1B02G283900  TraesCS1D02G274400 | 106 |

**
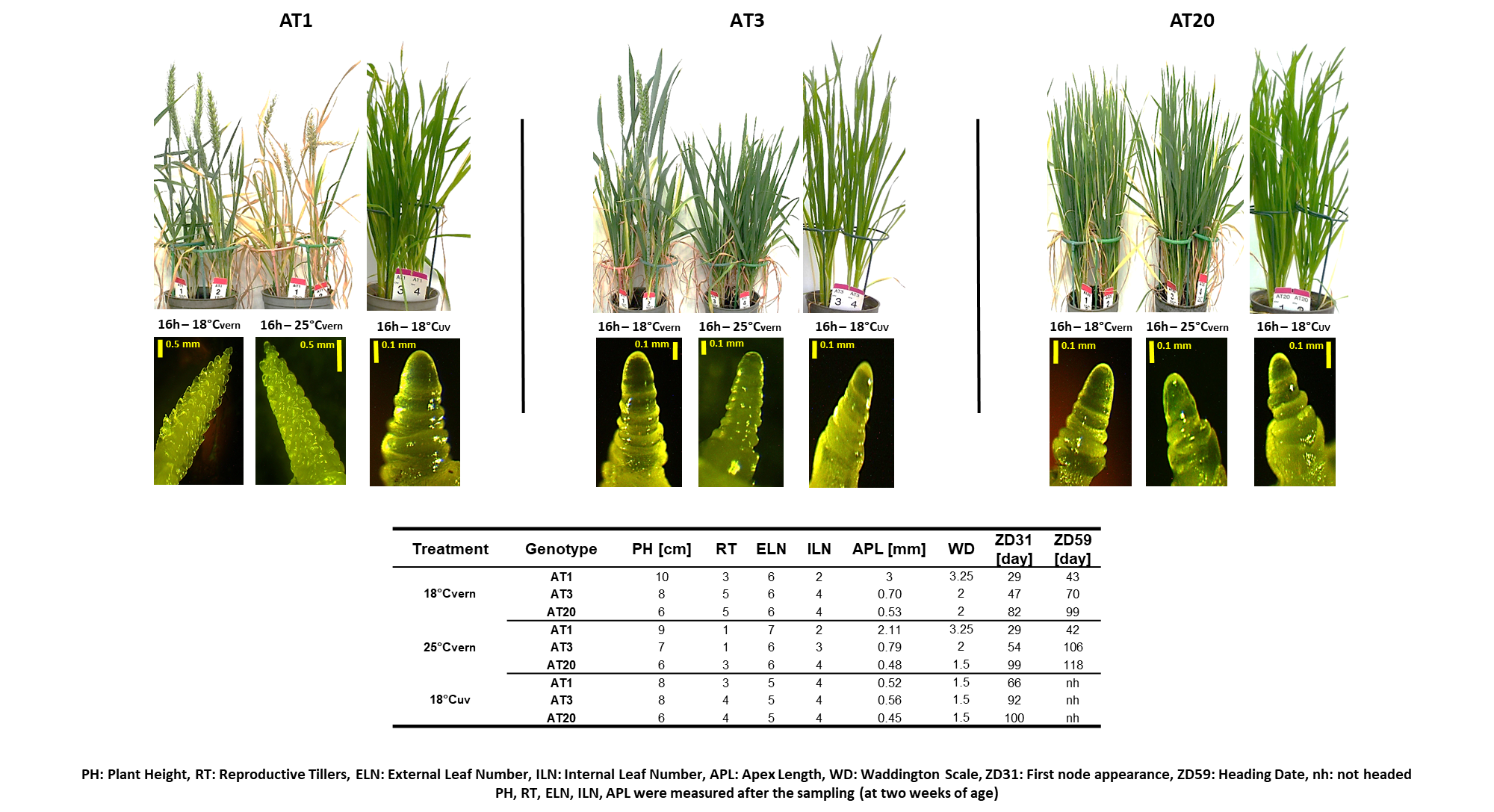
**

**Figure S2.** Plant developmental charactersitics of the three winter wheat cultivars (AT1-ʻMv Toborzóʼ, AT3-ʻTommiʼ and AT20-ʻChargerʼ) under the three environments (16 h-18ºCvern, 16 h-25ºCvern and 16 h-18ºCuv). Upper row: photos of 77-day old plants; middle row: photos of 14-day old apices; table below: developmental data at 14th day and days to first node appearance and to heading.

**Table S3.** Variance components (σ^2^ (%)) of circadian, photoreceptor and plant developmental genes in winter wheat plants grown for 14 days (a) under the three controlled growth chamber environments (25 °C vernalized + 18 °C vernalized + 18 °C unvernalized); (b) under the vernalized + unvernalized environments; and (c) under 25 °C vernalized + 18 °C vernalized environments by using the MLM module of GenStat 18.0.

| 1. **25°Cvern+18°Cvern+18°Cuv** | | | | | | | | | | | | | | | | | | | | |
| --- | --- | --- | --- | --- | --- | --- | --- | --- | --- | --- | --- | --- | --- | --- | --- | --- | --- | --- | --- | --- |
|  |  | **Circadian genes** | | | | | | |  | | | | | |  | | | | |  |
| **Factors** | **dF** | Morning loop | | Evening loop | | | Central genes | | **Photoreceptors** | | | | | | **Developmental genes** | | | | |  |
|  |  | *CCA1* | *PRR95* | *TOC1* | *LUX* | *ELF3* | *CO1* | *GI* | *PHYA* | *PHYB* | *PHYC* | *CRY1* | *CRY2* | *VRN1* | | *VRN2* | *VRN3* | *PPD1* |  |  |
| Genotype (G) | 2 | 4.0 | 3.6 | 9.0 | 0.6^ns^ | 9.2 | **40.0** | 9.8 | 1.2 | 1.4 | **12.9** | 0.3^ns^ | **55.0** | **61.5** | | **21.7** | **72.1** | 3.2 |  |  |
| Environment (E) | 2 | 8.1 | **29.4** | 3.7 | **11.0** | 5.4^**^ | **26.9** | **52.3** | **85.9** | **89.5** | **13.2** | **78.3** | 9.1 | **24.6** | | **56.3** | **11.2** | **71.8** |  |  |
| Daily Timing (DT) | 7 | **78.7** | **51.4** | **73.0** | **73.7** | **30.7** | 6.4 | **22.8** | 2.5 | 2.1 | **40.3** | 6.1 | **24.6** | 2.6 | | 6.8 | 1.1 | 9.3 |  |  |
| G × E | 4 | 5.3 | 2.3 | 1.2 | 8.5 | **12.1** | **19.4** | 9.7 | 1.3 | 1.0 | **13.1** | 8.0 | 1.7 | 8.8 | | 8.7 | 9.6 | 8.3 |  |  |
| G × DT | 14 | 1.0 | 2.8 | 3.0 | 0.8 | **20.2** | 3.5 | 0.9 | 3.9 | 2.5 | 8.5 | 3.0 | 3.1 | 1.1 | | 1.3 | 1.1 | 1.6 |  |  |
| E × DT | 14 | 1.8 | 9.0 | 8.2 | 2.8 | 7.5 | 2.3 | 2.9 | 2.5 | 1.5 | 5.2 | 2.0 | 4.4 | 0.8 | | 4.1 | 2.4 | 3.6 |  |  |
| G × E × DT | 28 | 1.0 | 1.5 | 1.6 | 2.5 | **13.6** | 1.1 | 1.4 | 2.5 | 1.7 | 4.8 | 2.2 | 1.6 | 0.5 | | 1.0 | 2.4 | 1.7 |  |  |
| Replication^1^ | 11 | 0.2^ns^ | 0.1^ns^ | 0.4^ns^ | 0.1^ns^ | 1.4^ns^ | 0.3^***^ | 0.2^ns^ | 0.2^*^ | 0.2^ns^ | 1.9 | 0.1^ns^ | 0.6^**^ | 0.1^ns^ | | 0.1^**^ | 0.1^ns^ | 0.5^**^ |  |  |
|  |  |  |  |  |  |  |  |  |  |  |  |  |  |  | |  |  |  |  |  |
| 1. **18°Cvern+18°Cuv** | | | | | | | | | | | | | | | | | | | | |
| Genotype (G) | 2 | **15.2** | 7.8 | **11.7** | 5.3 | **10.0** | **11.7** | **23.7** | 2.6 | 1.7^**^ | **13.2** | 4.3 | **45.8** | **24.4** | | **14.6** | **39.2** | 8.7 |  |  |
| Vernalization (V) | 1 | 1.8^**^ | 0.8^*^ | **10.3** | 0.7^ns^ | 8.1^**^ | **25.9** | 0.7^ns^ | **71.4** | **80.6** | **31.5** | **51.4** | **24.0** | **58.5** | | **52.4** | **32.8** | **54.3** |  |  |
| Daily Timing (DT) | 7 | **71.0** | **81.0** | **53.5** | **77.9** | **19.0** | **21.6** | **44.7** | 5.8 | 4.4 | **25.1** | 9.1 | **15.9** | 2.2 | | 6.2 | 2.6 | **15.7** |  |  |
| G × V | 2 | 3.2 | 2.4 | 1.3^*^ | 3.8 | 3.7^*^ | **14.6** | 23.5 | 2.6 | 2.6 | 4.1^**^ | **18.9** | 0.5^ns^ | **12.7** | | **17.5** | **19.9** | **11.4** |  |  |
| G × DT | 14 | 2.6 | 5.1 | 5.9 | 3.9 | **36.4** | **11.1** | 2.6 | 8.8 | 5.6 | **14.3** | 6.7 | 3.1 | 0.8 | | 1.0 | 2.6 | 3.5 |  |  |
| V × DT | 7 | 4.7 | 0.7^**^ | **15.4** | 4.7 | **12.8** | **12.0** | 0.5^ns^ | 4.7 | 2.6 | 5.9 | 4.9 | 8.5 | 1.0 | | 7.1 | 1.3 | 3.3 |  |  |
| G × V × DT | 14 | 0.8 | 1.9 | 1.1 | 3.4 | 8.4 | 2.8 | 3.6 | 3.6 | 2.2 | 4.1 | 4.5 | 1.5 | 0.4 | | 1.0 | 1.3 | 2.3 |  |  |
| Replication | 11 | 0.6^**^ | 0.3^ns^ | 0.7^*^ | 0.2^ns^ | 1.6^ns^ | 0.2^ns^ | 0.6^**^ | 0.6^*^ | 0.4^ns^ | 1.6^*^ | 0.2^ns^ | 0.6^ns^ | 0.1^ns^ | | 0.2^ns^ | 0.1^ns^ | 1.0 |  |  |
|  |  |  |  |  |  |  |  |  |  |  |  |  |  |  | |  |  |  |  |  |
| 1. **25°Cvern+18°Cvern** | | | | | | | | | | | | | | | | | | | | |
| Genotype (G) | 2 | 4.2 | 1.7 | 8.1 | 1.8 | 8.2 | **37.0** | **11.8** | 1.0 | 1.2 | **18.3** | 2.3 | **58.3** | **86.5** | | **55.2** | **87.3** | 3.6 |  |  |
| Temperature (T) | 1 | **17.6** | **48.0** | 2.2** | **20.7** | 6.7^*^ | **36.7** | **61.7** | **89.5** | **92.7** | 4.8^*^ | **76.0** | 6.6 | 4.4 | | **29.8** | 0.2^ns^ | **78.5** |  |  |
| Daily Timing (DT) | 7 | **62.9** | **30.1** | **80.8** | **55.7** | **27.0** | 3.7 | **11.0** | 1.8 | 1.4 | **41.6** | 3.7 | **19.3** | 3.5 | | 3.2 | 1.3 | 4.6 |  |  |
| G × T | 2 | **11.3** | 1.8 | 0.8^ns^ | **14.4** | **12.8** | **17.6** | 9.5 | 1.0 | 0.4^*^ | **14.4** | **12.3** | 2.8 | 2.0 | | 6.1 | 1.0 | 6.8 |  |  |
| G × DT | 14 | 1.1 | 2.5 | 3.5 | 1.2 | **17.4** | 2.4 | 1.2 | 2.4 | 1.6 | 7.4 | 2.4 | 3.1 | 1.6 | | 2.0 | 1.3 | 1.1 |  |  |
| T × DT | 7 | 1.0 | **14.1** | 1.5 | 3.6 | 4.2 | 1.2 | 3.3 | 1.9 | 1.1 | 4.1 | 1.3 | 7.0 | 1.2 | | 1.9 | 4.5 | 3.6 |  |  |
| G × T × DT | 14 | 1.7 | 1.8 | 2.6 | 2.5 | **22.7** | 1.2 | 1.2 | 2.3 | 1.5 | 7.5 | 2.0 | 2.2 | 0.7 | | 1.5 | 4.5 | 1.4 |  |  |
| Replication | 11 | 0.2^ns^ | 0.1^ns^ | 0.6^ns^ | 0.1^ns^ | 1.0^ns^ | 0.3 | 0.2^**^ | 0.1^*^ | 0.1^ns^ | 2.0^**^ | 0.1^ns^ | 0.8^**^ | 0.1^ns^ | | 0.3 | 0.1^ns^ | 0.3^**^ |  |  |

^1^ replication represents the same timepoint between the two days

Factors were signed with ns (not significant), **(P ≤ 0.01) or *(P ≤ 0.05) in the superscript. All others are higly significant (P ≤ 0.001)

Abbreviations: *CCA1* – *CIRCADIAN CLOCK-ASSOCIATED 1*, *CO1* – *CONSTANS 1*, *CRY1* – *CRYPTOCHROME 1*, *CRY2* – *CRYPTOCHROME 2*, *ELF3* – *EARLY FLOWERING 3*, *GI* – *GIGANTEA*, *LUX* – *ARRHYTHMO*, *PHYA* – *PHYTOCHROME A*, *PHYB* – *PHYTOCHROME B*, *PHYC* – *PHYTOCHROME C*, *PPD1* – *PHOTOPERIOD1*, *PRR95* – *PSEUDORESPONSE REGULATOR 95*, *TOC1* – *TIMING OF CAB EXPRESSION1*, *VRN1* – *VERNALIZATION1* (*APETALA1*), *VRN2* – *VERNALIZATION2*, *VRN3* – *VERNALIZATION3* (*FLOWERING LOCUS T*)

**Table S4.** Characteristics in daily expression patterns of circadian, photoreceptor and plant developmental genes in 14-day old plants of three winter wheat genotypes grown under three different environments.

Abbreviations: *CCA1* – *CIRCADIAN CLOCK-ASSOCIATED 1*, *CO1* – *CONSTANS 1*, *CRY1* – *CRYPTOCHROME 1*, *CRY2* – *CRYPTOCHROME 2*, *ELF3* – *EARLY FLOWERING 3*, *GI* – *GIGANTEA*, *LUX* – *ARRHYTHMO*, *PHYA* – *PHYTOCHROME A*, *PHYB* – *PHYTOCHROME B*, *PHYC* – *PHYTOCHROME C*, *PPD1* – *PHOTOPERIOD1*, *PRR95* – *PSEUDORESPONSE REGULATOR 95*, *TOC1* – *TIMING OF CAB EXPRESSION1*, *VRN1* – *VERNALIZATION1* (*APETALA1*), *VRN2* – *VERNALIZATION2*, *VRN3* – *VERNALIZATION3* (*FLOWERING LOCUS T*)

| **Genes** | | | **Treat.** | **AT1 (ʻMv Toborzóʼ)** | | | | | **AT3 (ʻTommiʼ)** | | | **AT20 (ʻChargerʼ)** | | | |
| --- | --- | --- | --- | --- | --- | --- | --- | --- | --- | --- | --- | --- | --- | --- | --- |
|  |  |  |  | **interval** | **min/max ratio** | | **Peak (hour of the day)** | | **interval** | **min/max ratio** | **Peak (hour of the day)** | **interval** | **min/max ratio** | **Peak (hour of the day)** | |
| **Circadian** | Morning loop | ***CCA1*** | 18°C | 0.48 – 20.3 | | 42.5 | | 9:00 | 0.02 – 8.94 | 445.2 | 6:00 | 0.2 – 8.26 | 42.2 | 6:00 – 9:00 |  |
|  |  |  | 25°C | 0.03 – 8.68 | | 277.7 | | 6:00 | 0.01 – 11.65 | 1568 | 6:00 | 0.03 – 8.95 | 262.7 | 6:00 |  |
|  |  |  | UV | 0.15 – 16.57 | | 111.5 | | 6:00 | 0.1 – 10.15 | 100 | 6:00 | 0.09 – 12.46 | 137.4 | 6:00 |  |
|  |  | ***PRR95*** | 18°C | 0.01 – 2.01 | | 150.3 | | 12:00 | 0.01 – 3.38 | 456.9 | 12:00 | 0.01 – 2.08 | 171.2 | 15:00 |  |
|  |  |  | 25°C | 0.002 – 0.91 | | 380.9 | | 15:00 | 0.003 – 0.58 | 216 | 15:00 | 0.002 – 1 | 596.2 | 15:00 |  |
|  |  |  | UV | 0.01 – 1.78 | | 159.6 | | 12:00 | 0.01 – 2.08 | 251.7 | 12:00 | 0.01 – 1.12 | 90 | 12:00 |  |
|  | Evening loop | ***TOC1*** | 18°C | 0.09 – 1.83 | | 20.7 | | 18:00; 24:00 – 3:00 | 0.12 – 1.99 | 16.4 | 21:00 | 0.11 – 1.71 | 15.1 | 18:00 |  |
|  |  |  | 25°C | 0.12 – 2.2 | | 18.4 | | 18:00 | 0.03 – 1.73 | 67.3 | 18:00 | 0.06 – 2.44 | 38.5 | 18:00 |  |
|  |  |  | UV | 0.1 – 2.78 | | 27.3 | | 15:00; 24:00 – 03:00 | 0.05 – 2.01 | 37.9 | 15:00 | 0.09 – 1.67 | 18.9 | 15:00 |  |
|  |  | ***LUX*** | 18°C | 0.21 – 6.98 | | 32.6 | | 18:00 | 0.07 – 9.28 | 126.1 | 15:00 | 0.21 – 10.29 | 48.5 | 15:00 |  |
|  |  |  | 25°C | 0.12 – 9.81 | | 82.5 | | 18:00 | 0.01 – 5.12 | 939.1 | 18:00 | 0.06 – 6.9 | 114.7 | 18:00 |  |
|  |  |  | UV | 0.16 – 5.31 | | 33.1 | | 15:00 – 18:00 | 0.08 – 9.3 | 117.5 | 18:00 | 0.03 – 5.95 | 175.3 | 21:00 |  |
|  |  | ***ELF3*** | 18°C | 0.02 – 2.14 | | 98.5 | | 3:00 | 0.05 – 0.76 | 15 | 9:00; 18:00 | 0.03 – 0.67 | 22.1 | 6:00; 18:00 – 21:00 |  |
|  |  |  | 25°C | 0.07 – 0.97 | | 13.7 | | - | 0.04 – 0.45 | 10.8 | 3:00 | 0.09 – 0.88 | 9.6 | 3:00 |  |
|  |  |  | UV | 0.04 – 1.1 | | 24.9 | | 15:00; 3:00 | 0.1 – 0.51 | 5 | 12:00 – 15:00; 24:00 | 0.04 – 0.45 | 11.2 | 12:00; 24:00 |  |
|  | Central | ***CO1*** | 18°C | 0.001 – 0.47 | | 777.8 | | 24:00 – 3:00 | 0.03 – 0.47 | 17.8 | 15:00 – 21:00 | 0.02 – 0.9 | 39.8 | 18:00 |  |
|  |  |  | 25°C | 0.03 – 0.95 | | 28.7 | | 6:00; 3:00 | 0.01 – 0.42 | 60.9 | 6:00; 18:00 | 0.13 – 1.58 | 12 | 15:00 – 18:00; 24:00 – 3:00 |  |
|  |  |  | UV | 0.04 – 0.57 | | 13.1 | | 6:00; 21:00 | 0.03 – 0.8 | 31.8 | 18:00 – 21:00 | 0.05 – 0.8 | 17.1 | 18:00 |  |
|  |  | ***GI*** | 18°C | 0.001 – 0.72 | | 958.3 | | 18:00 | 0.02 – 1.7 | 79.1 | 15:00 | 0.05 – 1.96 | 43.5 | 18:00 |  |
|  |  |  | 25°C | 0.001 – 0.43 | | 426.4 | | 18:00 | 0.003 – 0.18 | 56.5 | - | 0.003 – 0.56 | 197.4 | 15:00 – 18:00 |  |
|  |  |  | UV | 0.03 – 1.09 | | 41.9 | | 18:00 | 0.02 – 1.27 | 77 | 18:00 | 0.03 – 0.86 | 31.1 | 18:00 |  |
| **Photoreceptor** | | ***PHYA*** | 18°C | 0.07 – 1.91 | | 25.9 | | 24:00 | 0.06 – 0.89 | 15.7 | 6:00; 15:00 | 0.08 – 0.98 | 11.8 | 6:00; 12:00 – 18:00; 24:00 |  |
|  |  |  | 25°C | 0.005 – 0.08 | | 17.9 | | - | 0.003 – 0.09 | 31.5 | - | 0.01 – 0.14 | 13.1 | - |  |
|  |  |  | UV | 0.04 – 0.35 | | 9.4 | | - | 0.03 – 0.39 | 13.6 | - | 0.04 – 0.43 | 10.8 | - |  |
|  |  | ***PHYB*** | 18°C | 0.08 – 1.82 | | 23.7 | | 24:00 | 0.04 – 1.53 | 36.2 | 6:00; 15:00 | 0.11 – 1.52 | 14.3 | 6:00; 12:00 – 18:00; 24:00 |  |
|  |  |  | 25°C | 0.02 – 0.2 | | 9.6 | | - | 0.006 – 0.16 | 27.9 | - | 0.01 – 0.16 | 14.4 | - |  |
|  |  |  | UV | 0.07 – 0.53 | | 7.4 | | - | 0.03 – 0.38 | 13.5 | - | 0.02 – 0.54 | 23 | - |  |
|  |  | ***PHYC*** | 18°C | 0.04 – 1.23 | | 31.2 | | 3:00 | 0.05 – 0.65 | 13.7 | 12:00; 3:00 | 0.06 – 0.83 | 14.2 | 15:00 |  |
|  |  |  | 25°C | 0.06 – 0.7 | | 11.3 | | 3:00 | 0.02 – 0.49 | 30.4 | 3:00 | 0.08 – 0.97 | 12.2 | 3:00 |  |
|  |  |  | UV | 0.04 – 0.43 | | 10.4 | | - | 0.1 – 0.47 | 4.5 | - | 0.02 – 0.29 | 13.7 | - |  |
|  |  | ***CRY1*** | 18°C | 0.1 – 8.96 | | 86.1 | | 3:00 | 1.53 – 9.71 | 6.4 | 12:00; 24:00 | 1.04 – 7.69 | 7.4 | - |  |
|  |  |  | 25°C | 0.58 – 5.26 | | 9 | | 3:00 | 0.31 – 2.32 | 7.6 | 6:00 | 0.46 – 5.08 | 11.1 | 3:00 |  |
|  |  |  | UV | 1.78 – 18.66 | | 10.5 | | 6:00; 3:00 | 2.02 – 8.55 | 4.2 | 6:00; 21:00 | 0.91 – 10.59 | 11.6 | 6:00; 18:00; 3:00 |  |
|  |  | ***CRY2*** | 18°C | 0.15 – 2.35 | | 15.4 | | 12:00 | 0.27 – 2.69 | 9.9 | 12:00 | 0.18 – 1.68 | 9.6 | - |  |
|  |  |  | 25°C | 0.25 – 2.68 | | 10.7 | | 3:00 | 0.54 – 3.81 | 7 | 6:00; 3:00 | 0.39 – 2.49 | 6.4 | 6:00 |  |
|  |  |  | UV | 0.36 – 4.23 | | 11.8 | | 6:00; 3:00 | 0.84 – 3.35 | 4 | 6:00; 3:00 | 0.33 – 1.88 | 5.8 | 6:00; 15:00; 3:00 |  |
| **Developmental** | | ***VRN1*** | 18°C | 1.29 – 9 | | 7 | | 9:00 | 0.42 – 4.3 | 10.1 | 12:00 | 0.11 – 2.09 | 19.6 | 12:00 |  |
|  |  |  | 25°C | 1.28 – 8.15 | | 6.4 | | 18:00 | 0.14 – 2.54 | 18.5 | 12:00 | 0.05 – 0.88 | 16.7 | 9:00; 18:00 |  |
|  |  |  | UV | 0.02 – 0.21 | | 9.7 | | - | 0.02 – 0.28 | 13.9 | - | 0.02 – 0.35 | 18.2 | - |  |
|  |  | ***VRN2*** | 18°C | 0.15 – 0.95 | | 6.3 | | - | 0.16 – 2.26 | 14.1 | 6:00; 15:00 | 0.72 – 7.89 | 11 | 12:00 |  |
|  |  |  | 25°C | 0.02 – 0.62 | | 37.1 | | 6:00 | 0.005 – 0.89 | 186.2 | 6:00 | 0.03 – 7.29 | 222.7 | 6:00; 18:00 |  |
|  |  |  | UV | 0.25 – 9.8 | | 38.5 | | 6:00 – 9:00; 21:00 | 0.06 – 7.99 | 141.3 | 6:00; 18:00 – 21:00 | 0.15 – 8.06 | 54.6 | 6:00; 18:00 – 21:00 |  |
|  |  | ***VRN3*** | 18°C | 0.23 – 4.58 | | 19.6 | | 24:00 | 0.05 – 0.7 | 15.5 | 6:00 – 12:00; 24:00 | 0.001 – 0.53 | 453.5 | 12:00 |  |
|  |  |  | 25°C | 0.2 – 4.86 | | 24.2 | | 9:00; 15:00 | 0.002 – 0.53 | 219.6 | 6:00 | 0.002 – 0.08 | 51.2 | - |  |
|  |  |  | UV | 0.007 – 0.2 | | 27.3 | | - | 0.1 – 0.19 | 18.5 | 6:00 – 12:00; 24:00 | 0.004 – 0.22 | 50.9 | 6:00 – 9:00; 21:00 |  |
|  |  | ***PPD1*** | 18°C | 0.15 – 4.15 | | 27.7 | | 6:00; 18:00 | 0.19 – 5.15 | 27.7 | 6:00; 12:00; 24:00 | 0.09 – 10.09 | 116.7 | 6:00; 12:00; 24:00 |  |
|  |  |  | 25°C | 0.19 – 1.38 | | 7.4 | | 3:00 | 0.01 – 0.54 | 98.2 | - | 0.01 – 1.37 | 161.3 | - |  |
|  |  |  | UV | 0.28 – 2.3 | | 8.1 | | 6:00; 24:00 | 0.03 – 2.99 | 105.5 | 6:00; 12:00; 18:00 | 0.01 – 1.66 | 222.7 | 6:00; 18:00 |  |

**a**

**
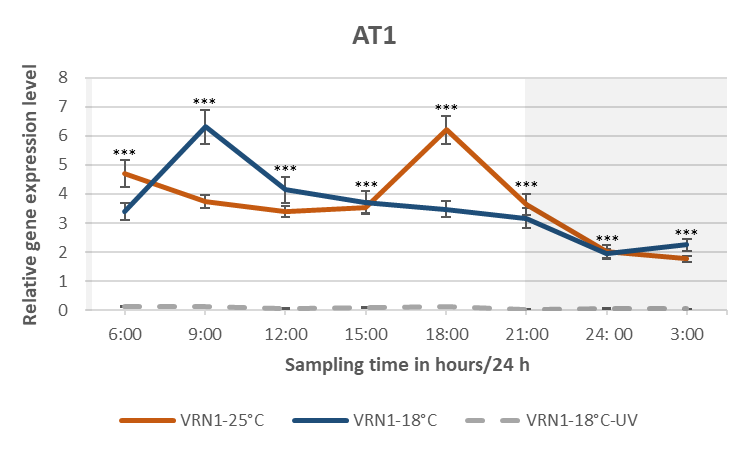

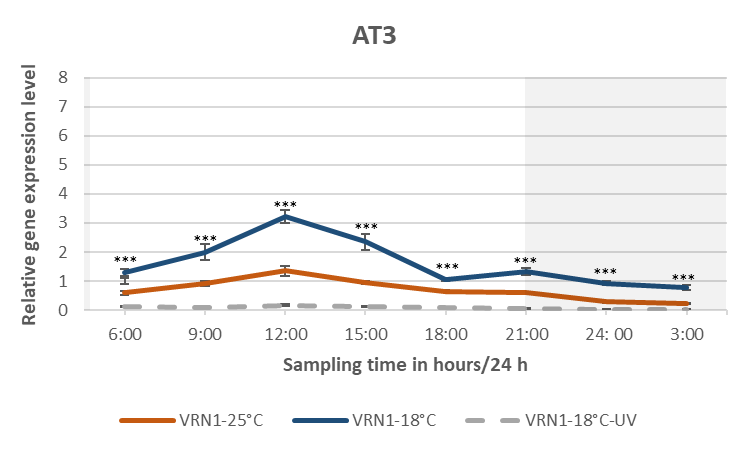
**

**
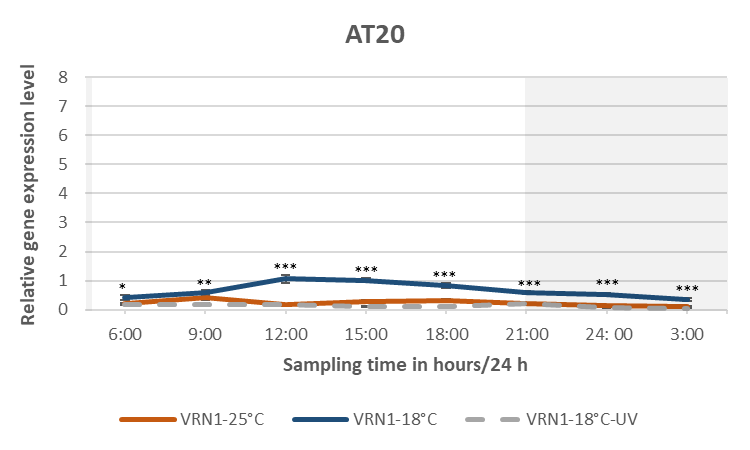
**

**b**


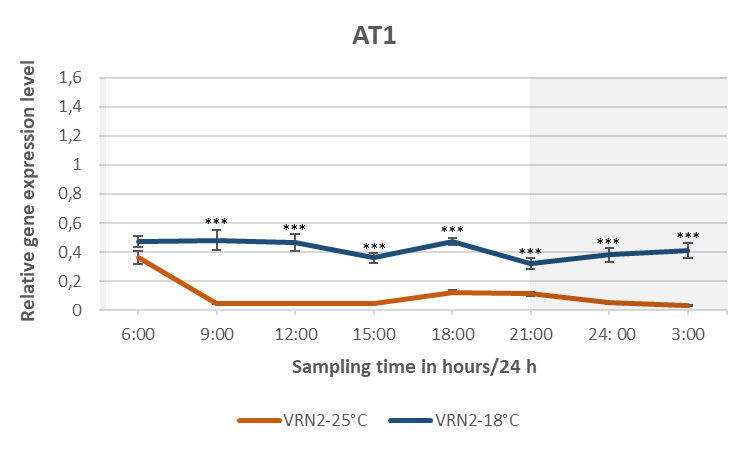

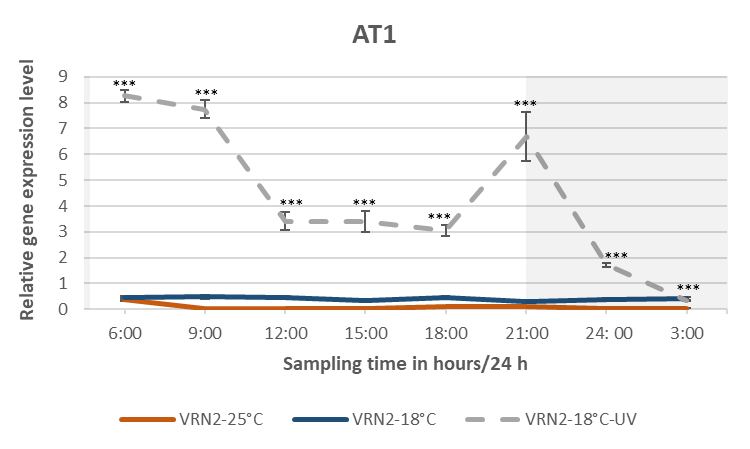


**
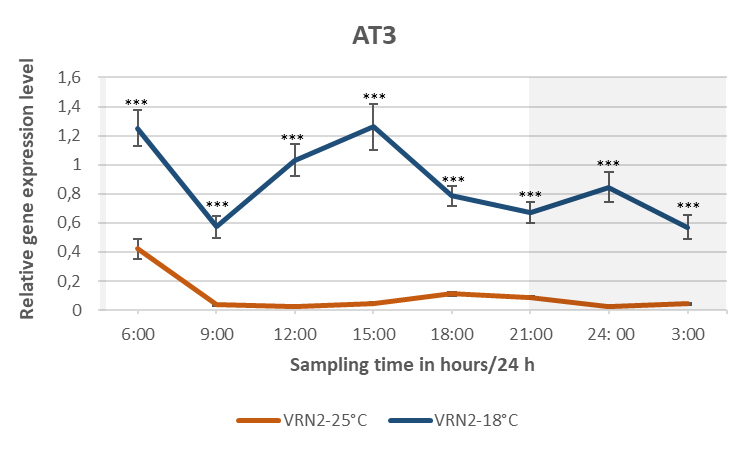

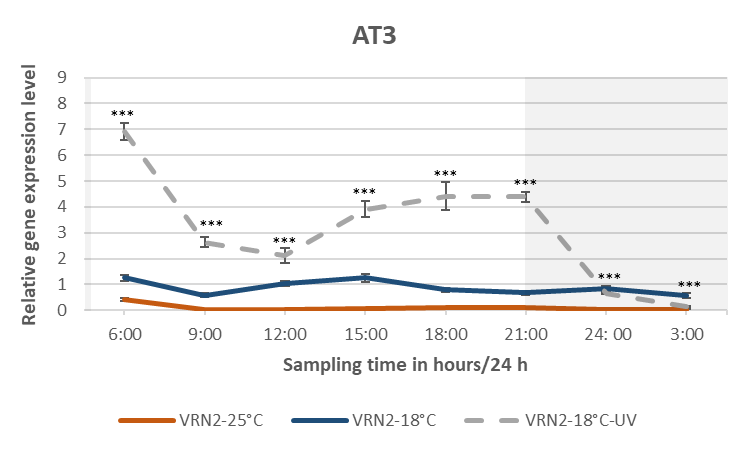
**

**
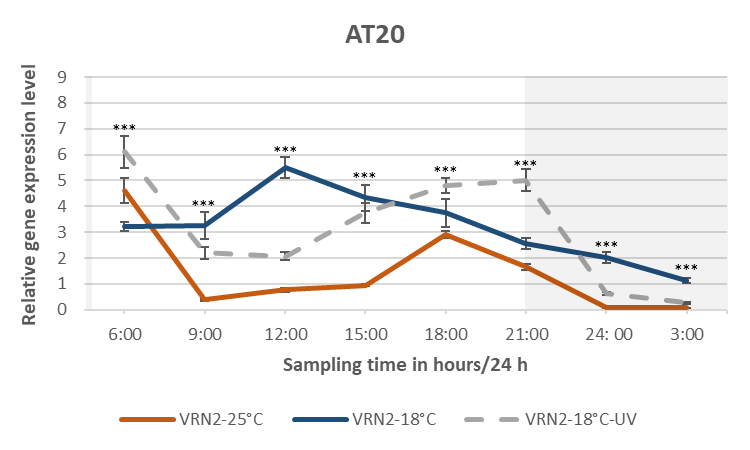
**

**c**


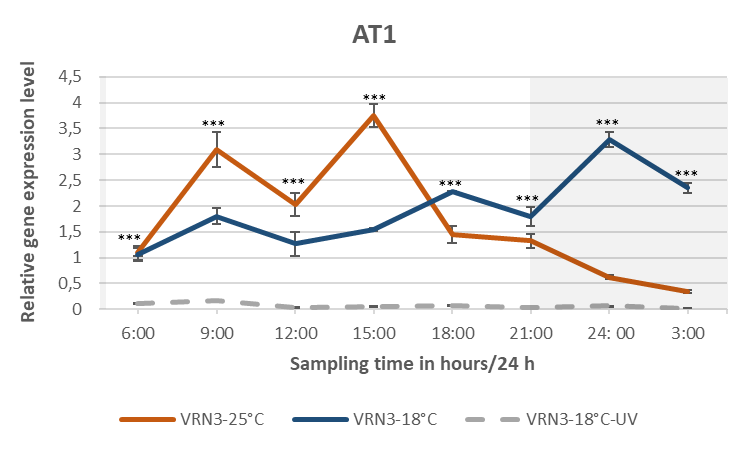


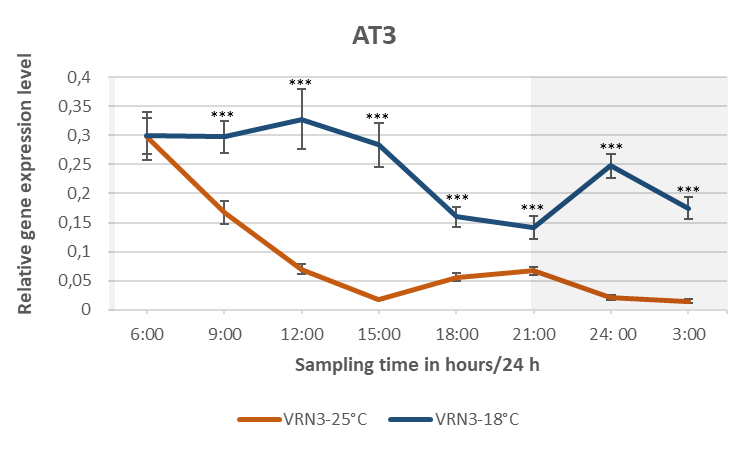

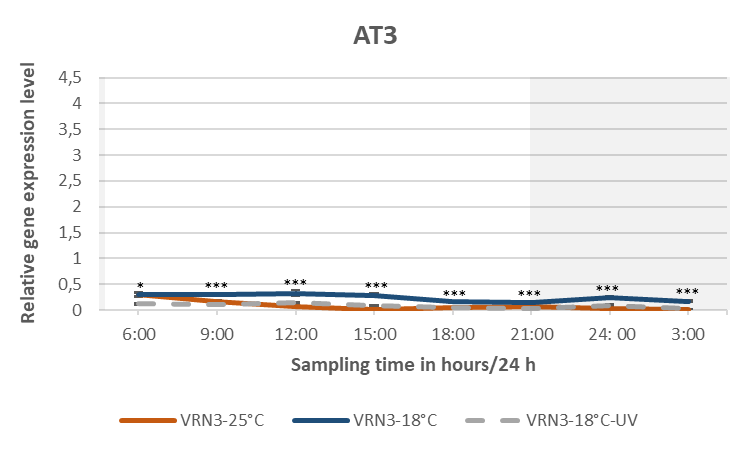


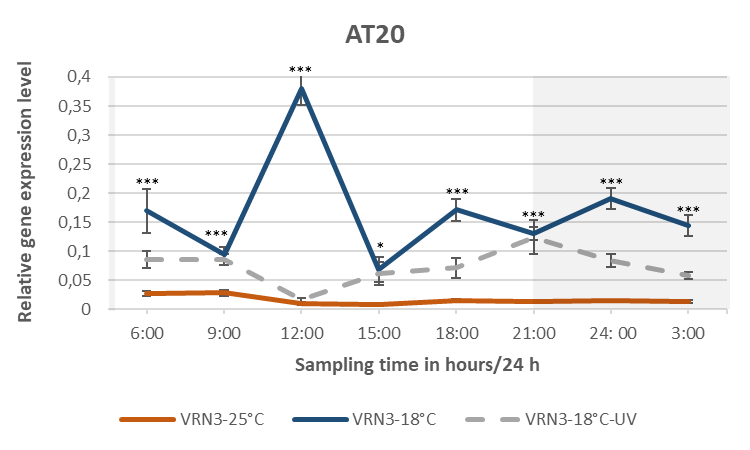

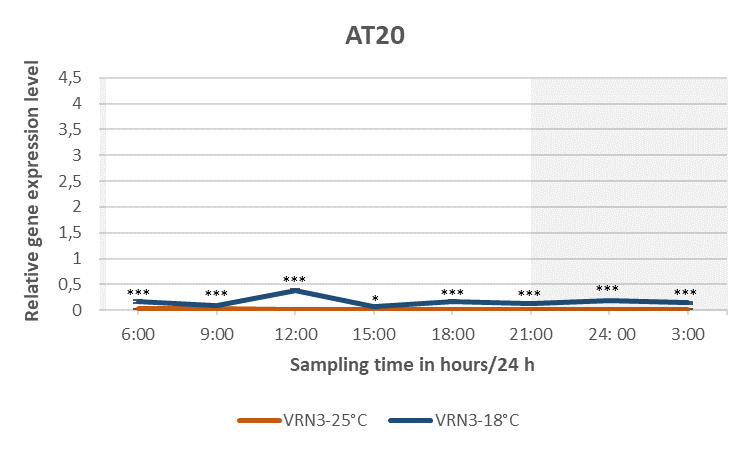


**d**


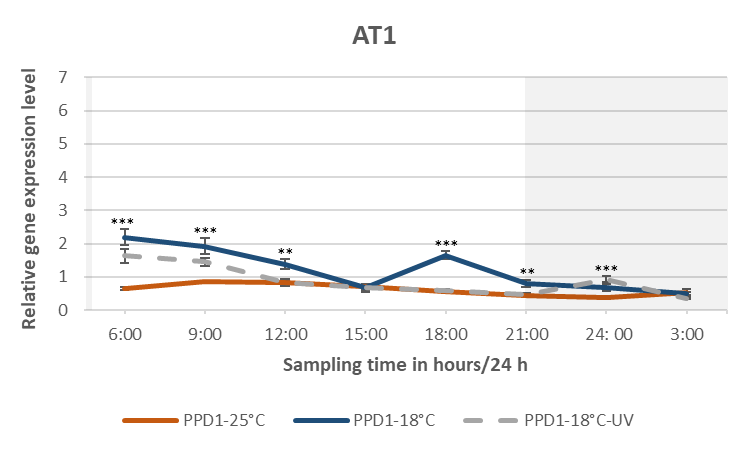

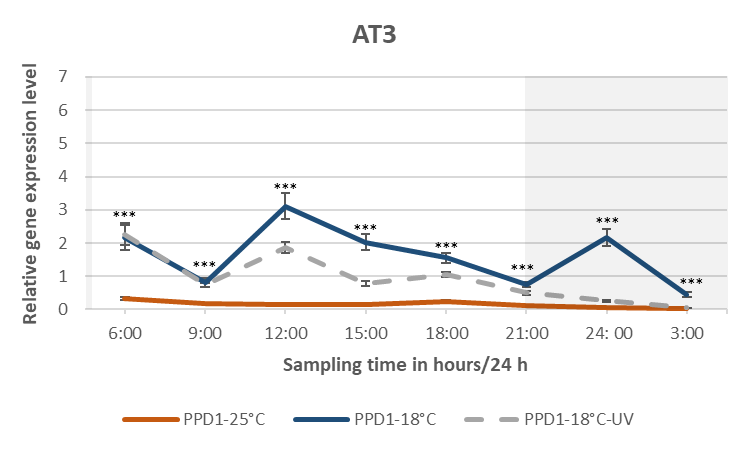


**
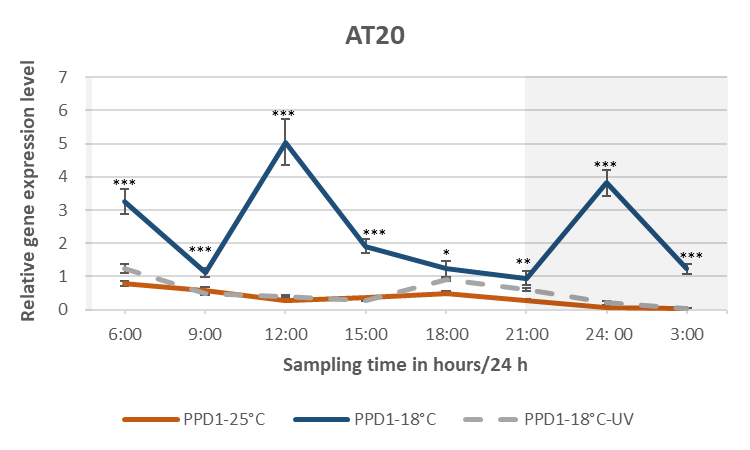
**

**e**


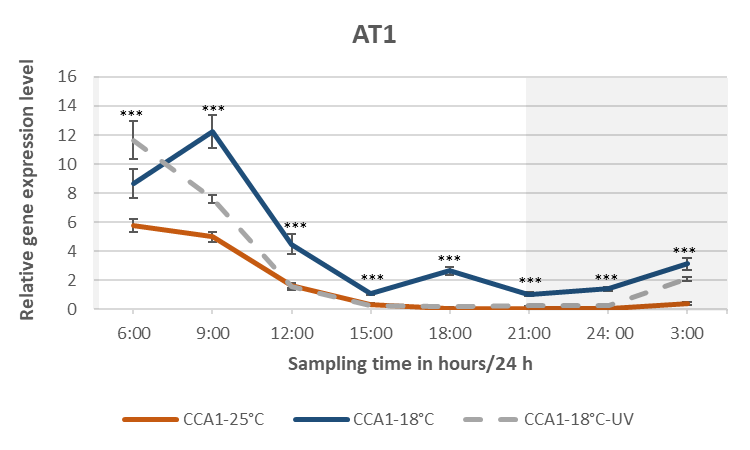

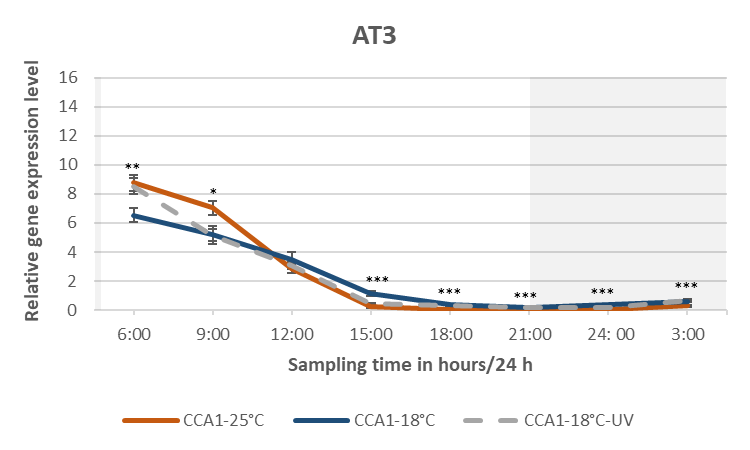


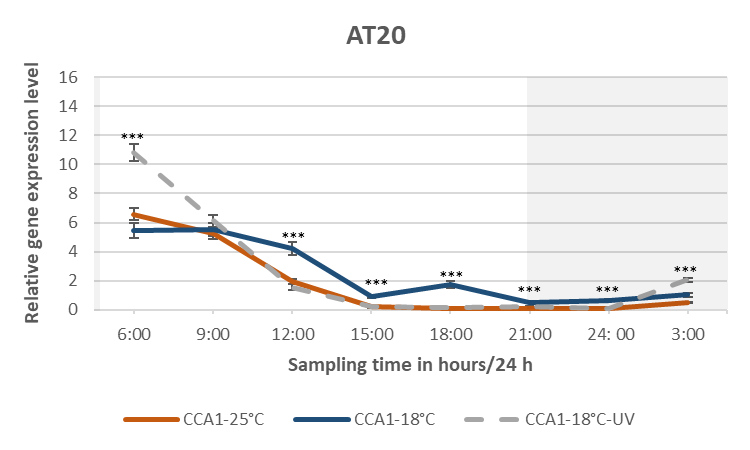


**f**


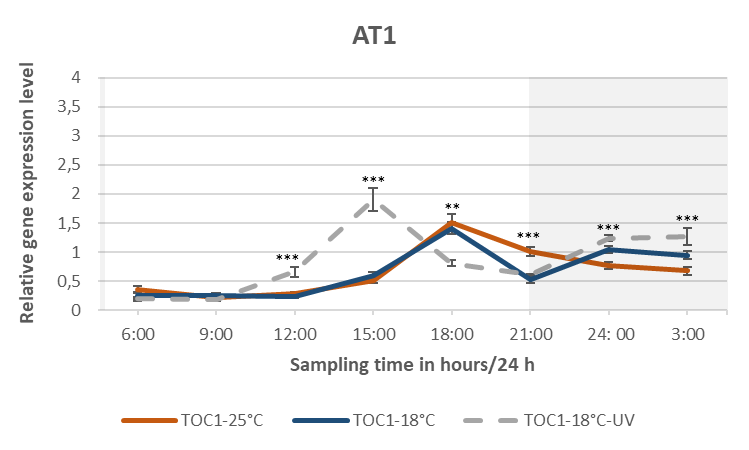

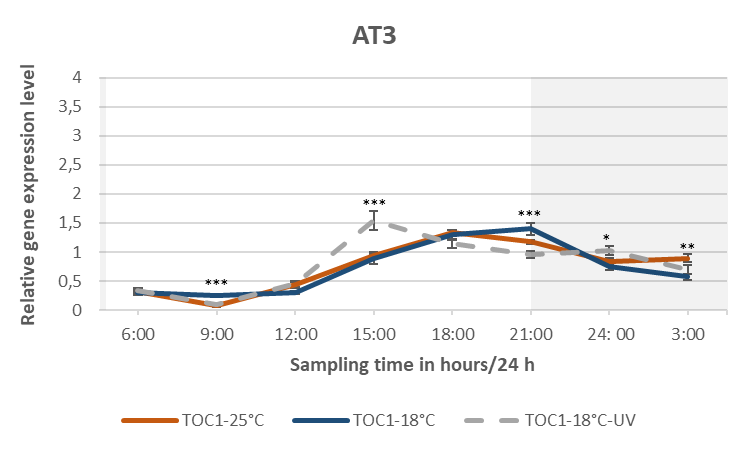


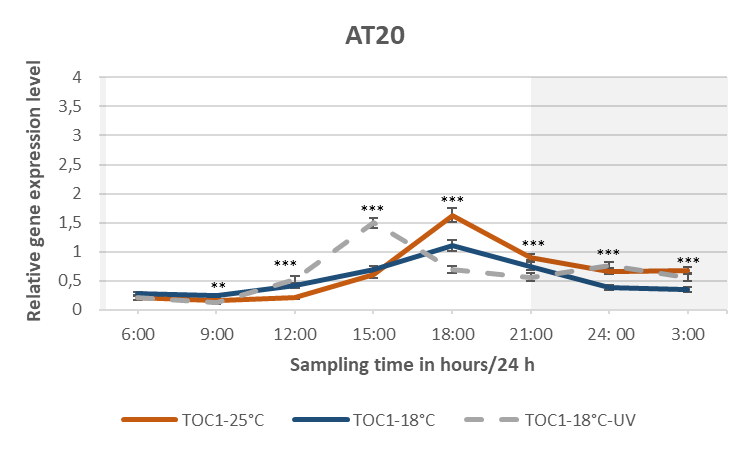


**g**


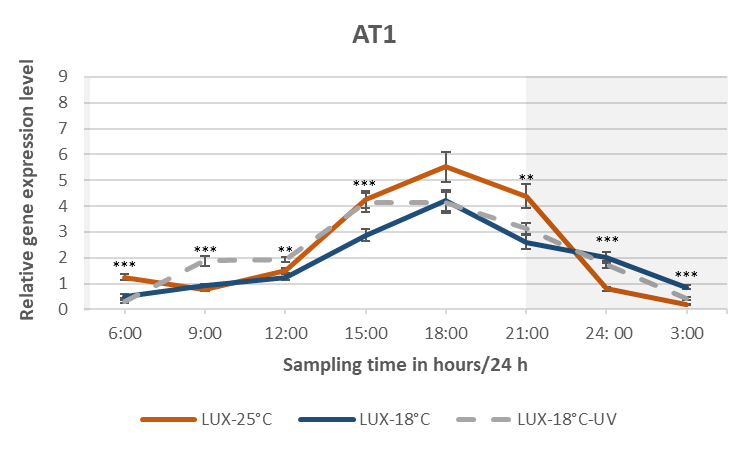

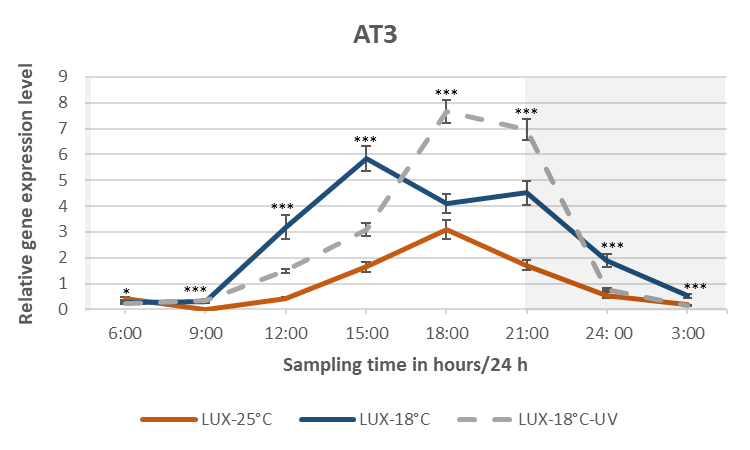


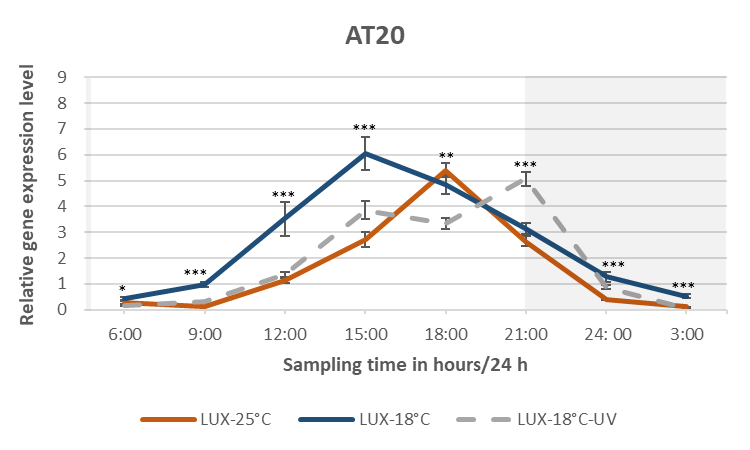


**h**


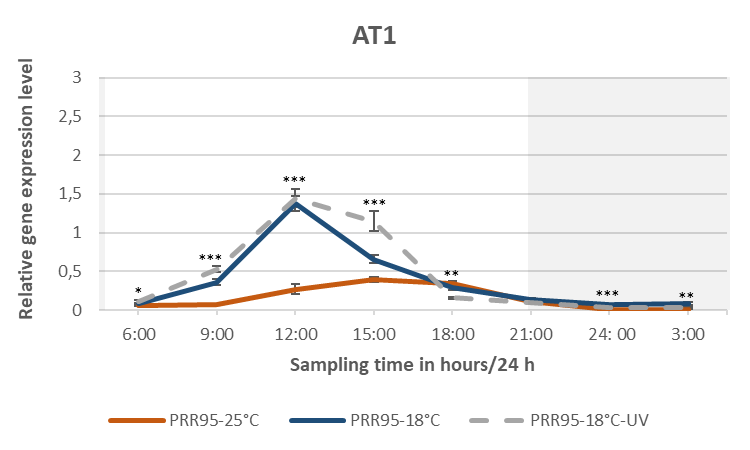

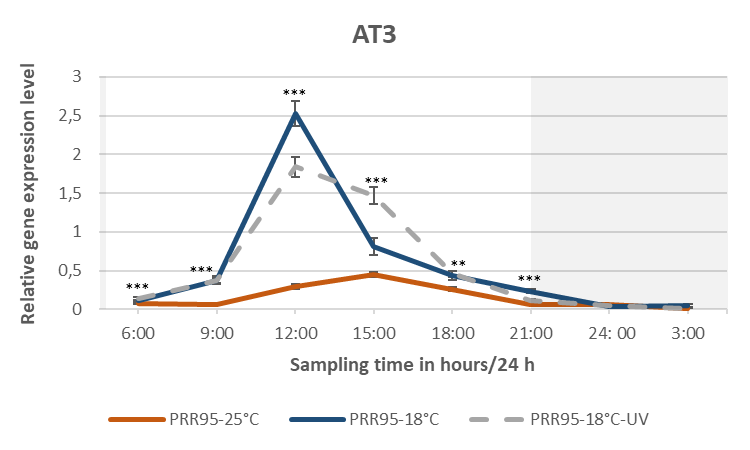


**
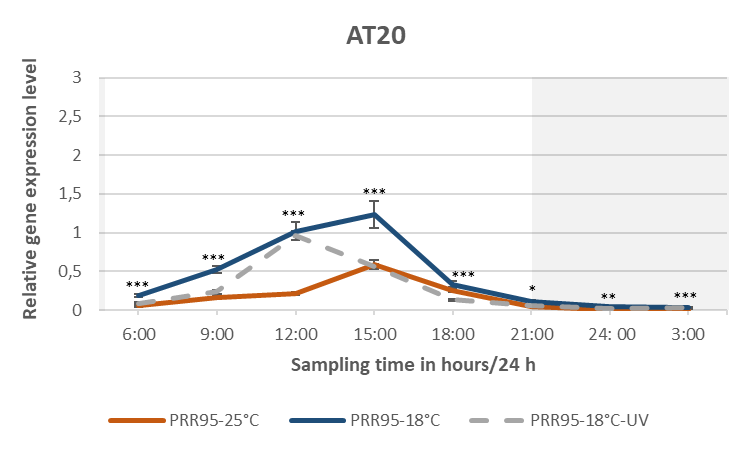
**

**i**


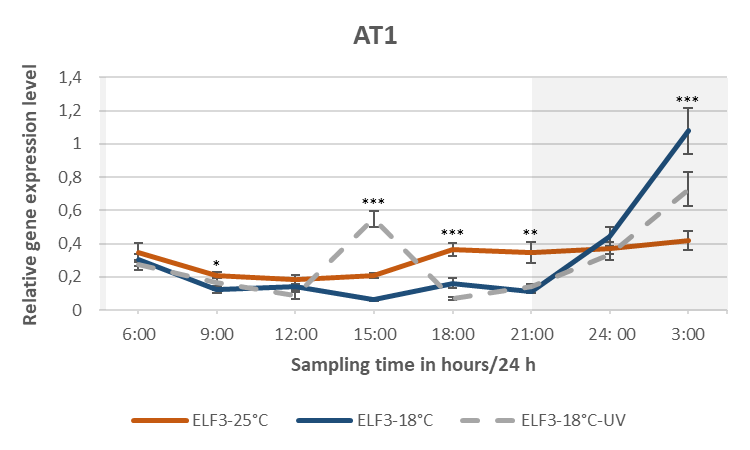

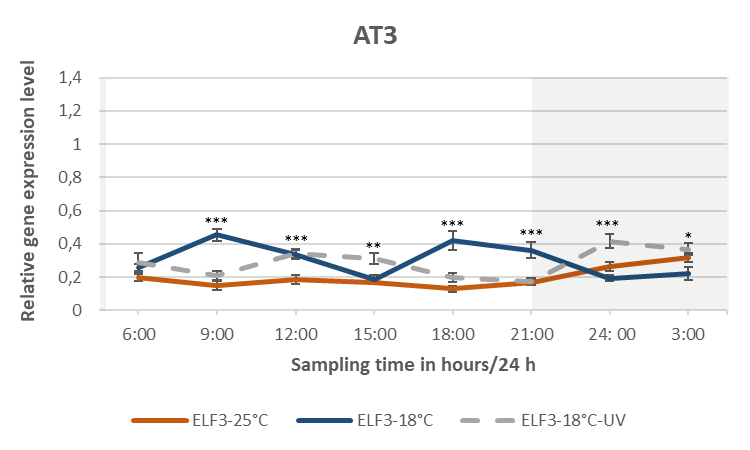


**
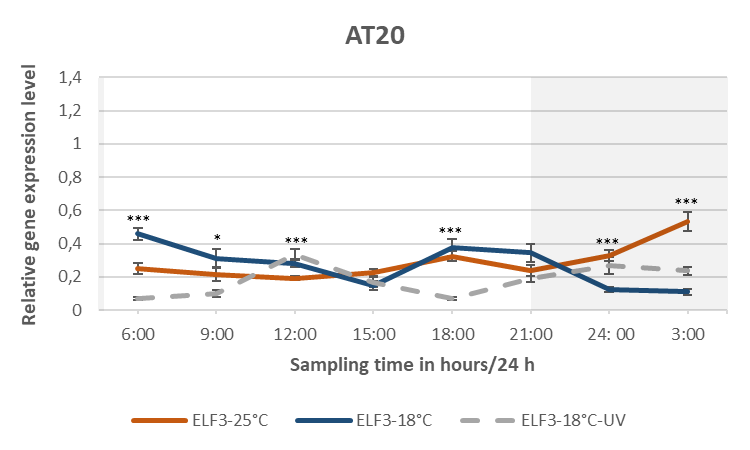
**

**j**


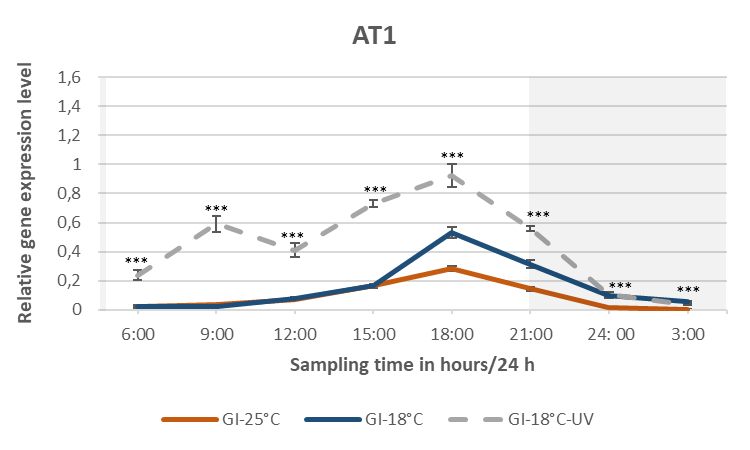

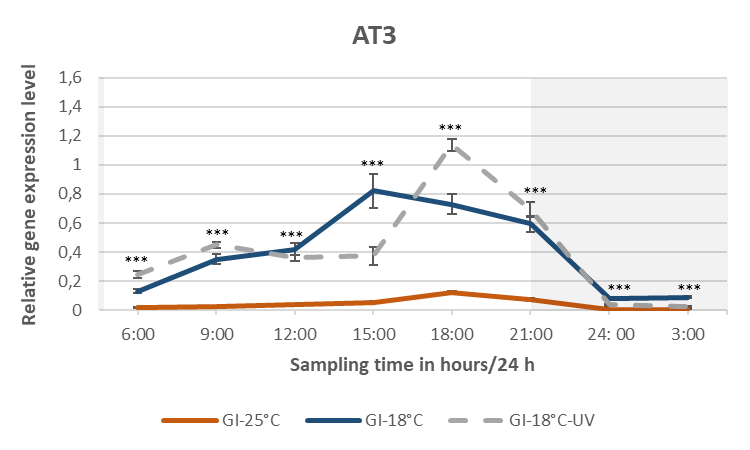


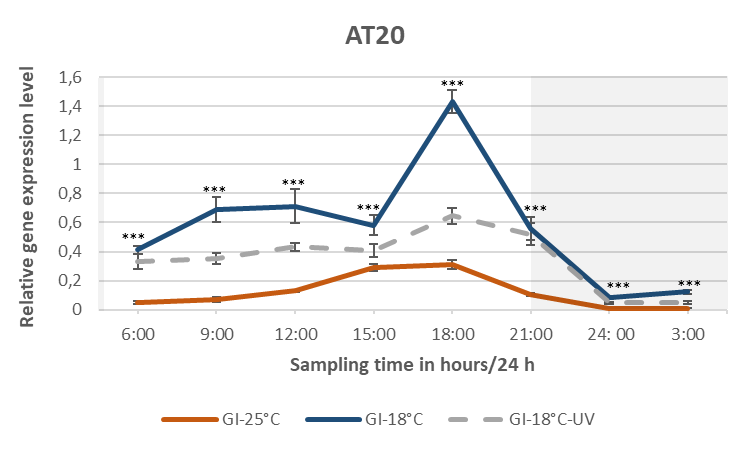


**k**


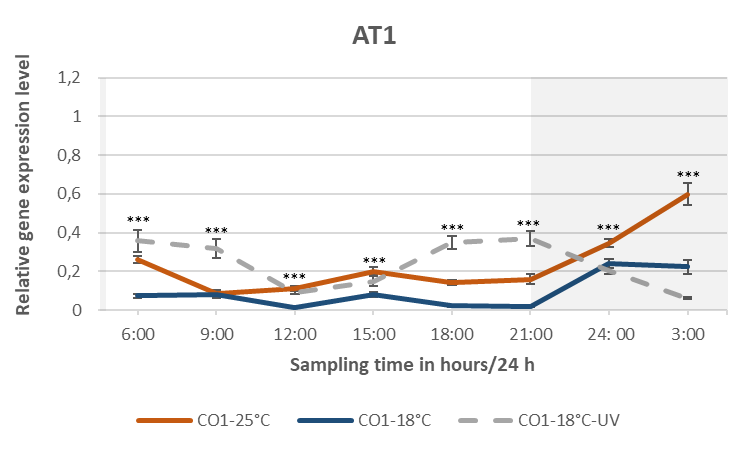

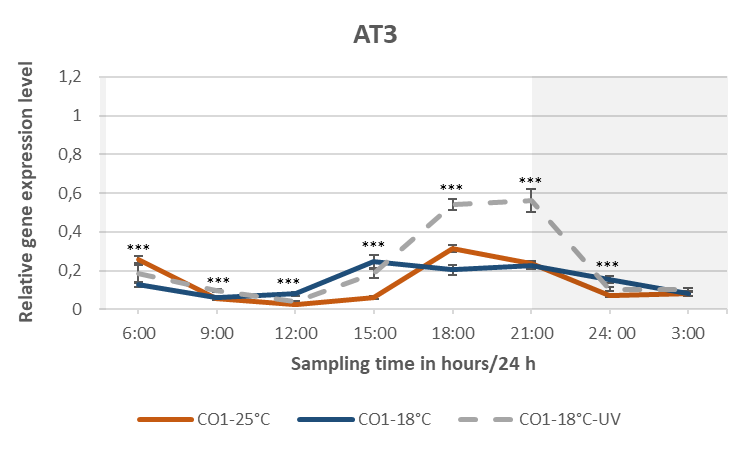


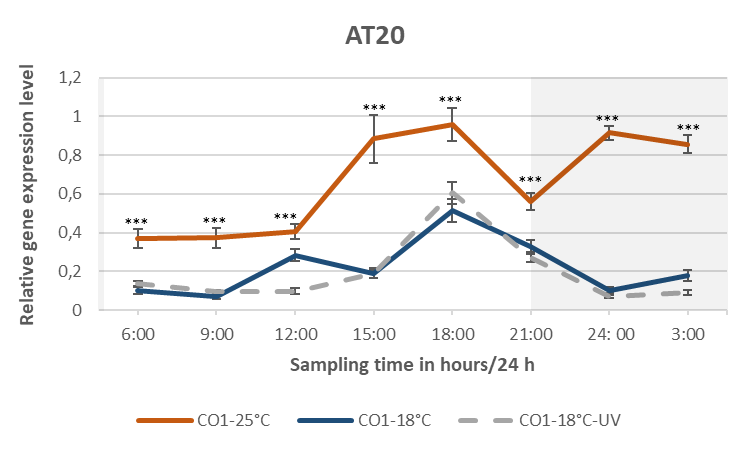


**l**


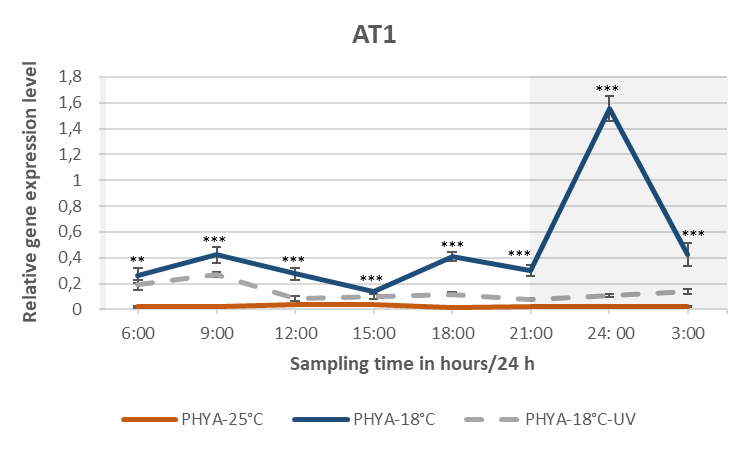

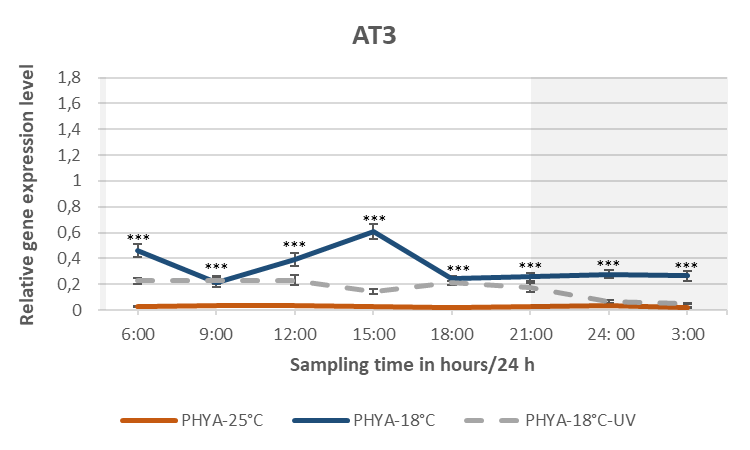


**
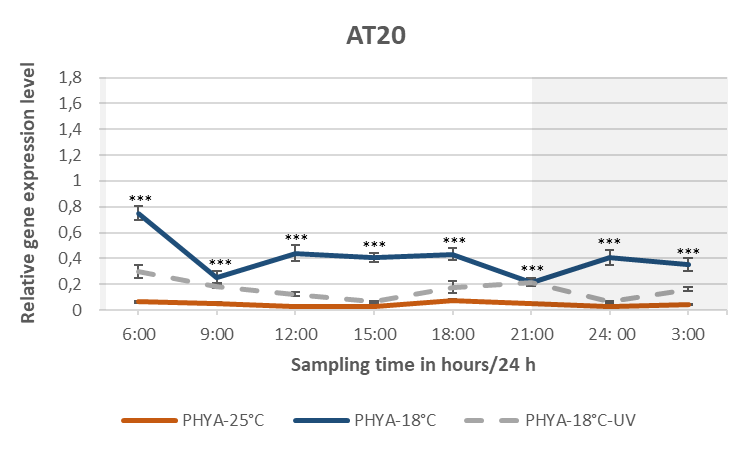
**

**m**


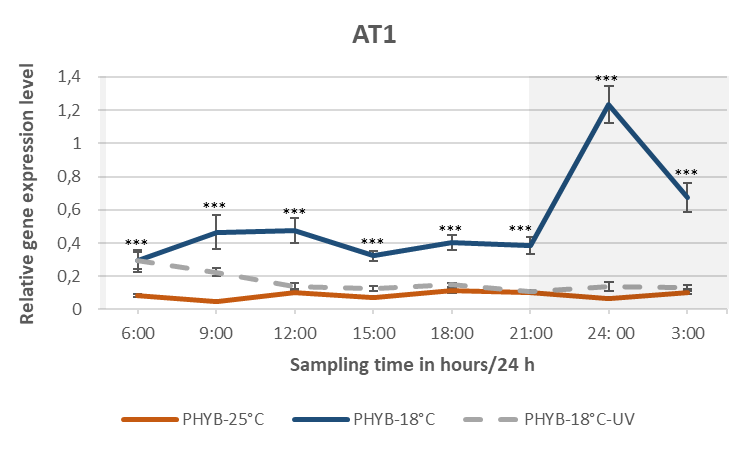

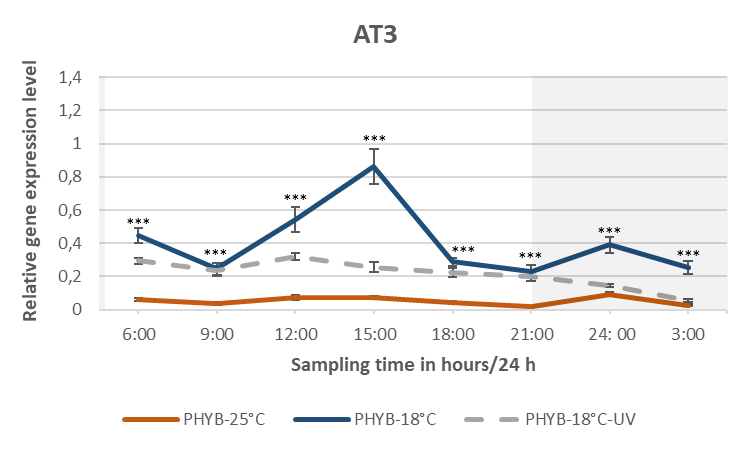


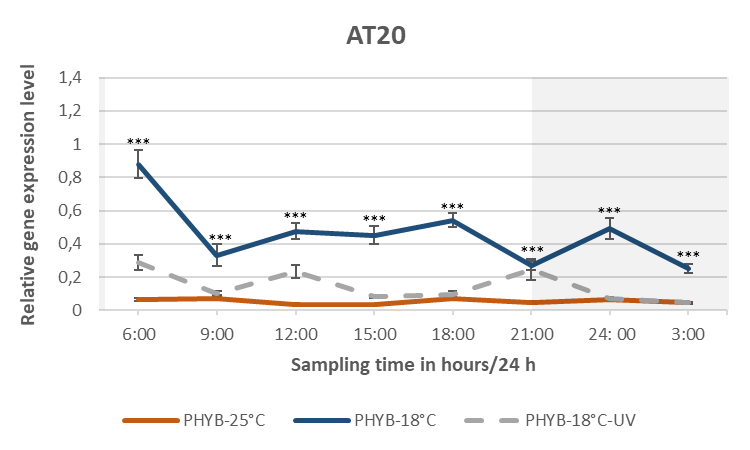


**n**


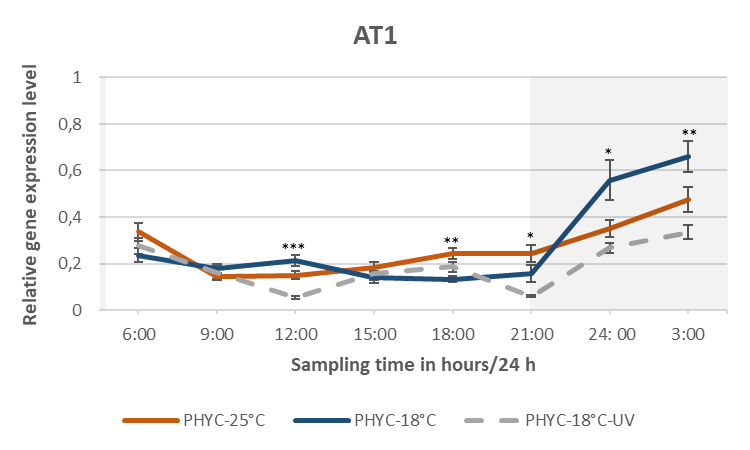

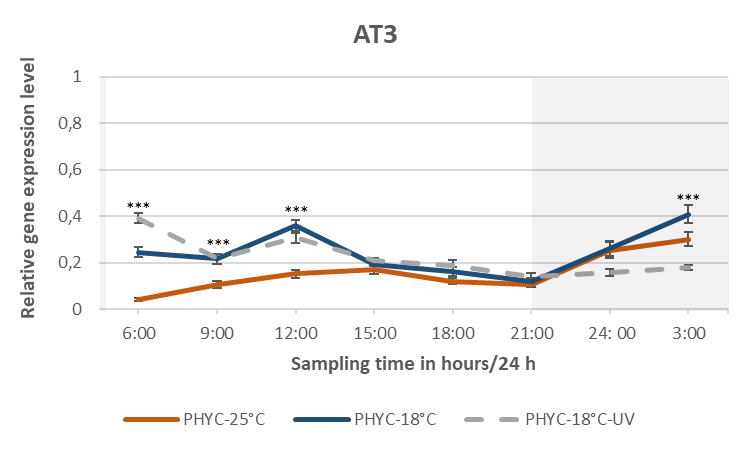


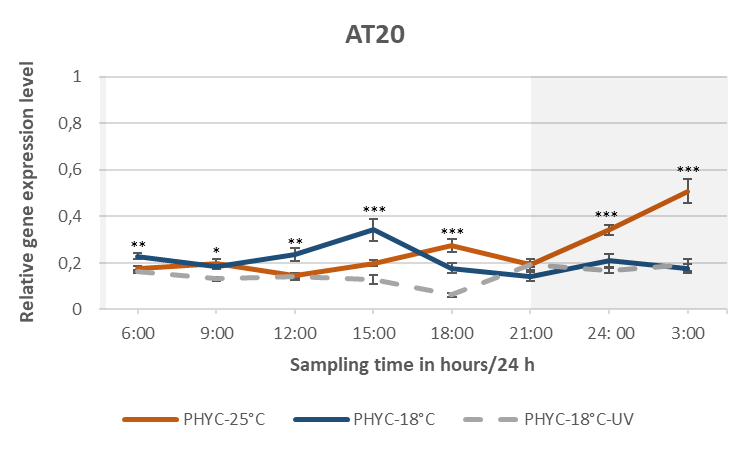


**o**


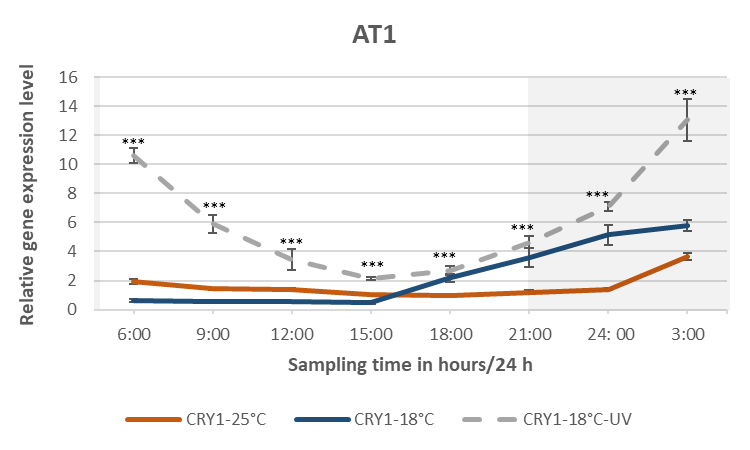

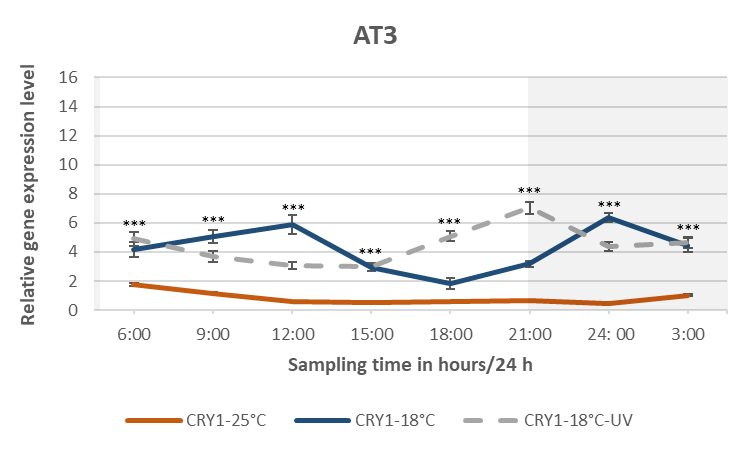


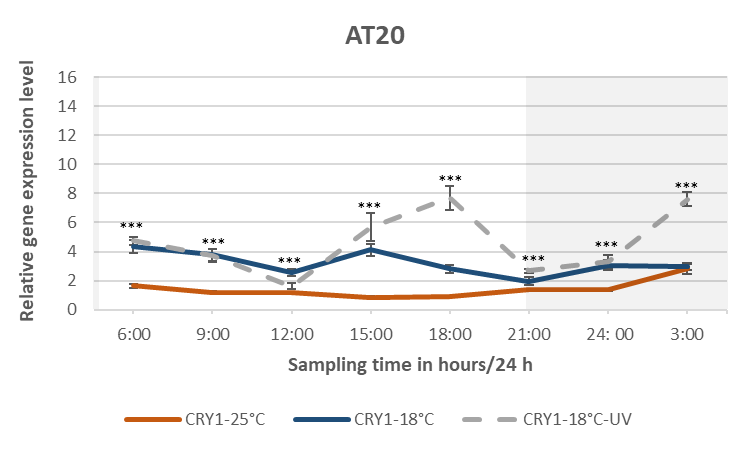


**p**


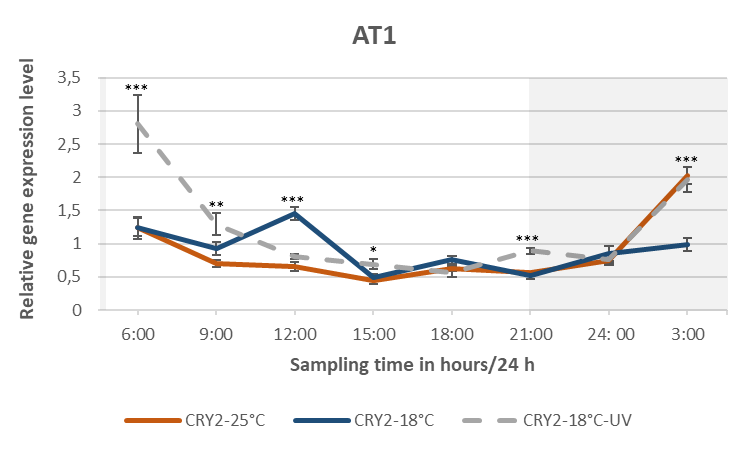

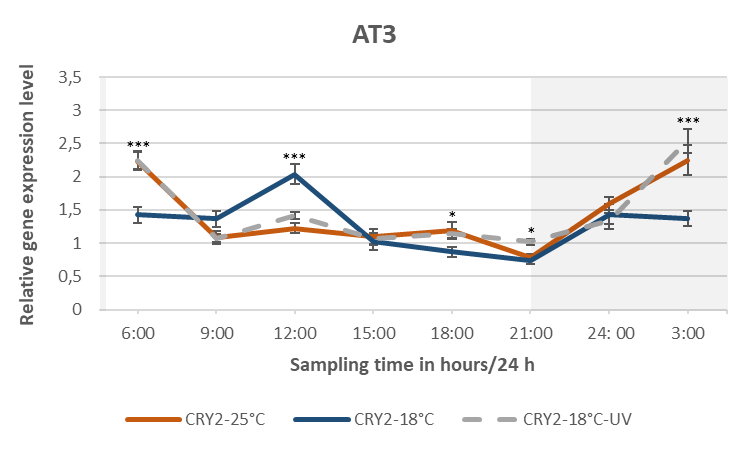


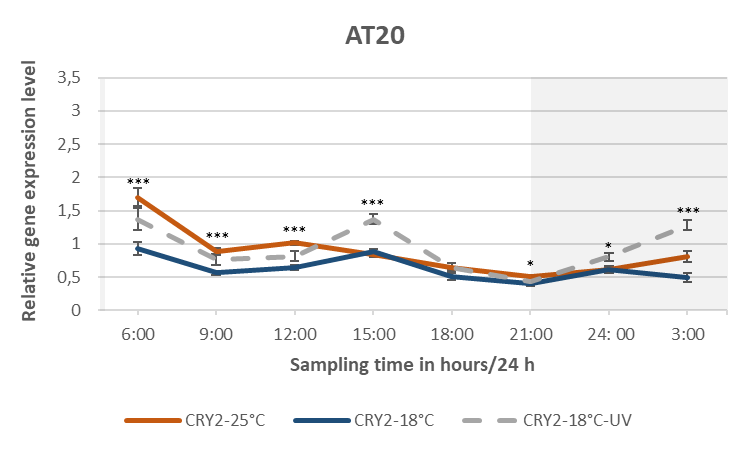


**Figure S3.** The daily dynamics in the expression levels of *VRN1* (a), *VRN2* (b), *VRN3* (c), *PPD1* (d), *CCA1* (e), *TOC1* (f), *LUX* (g), *PRR95* (h), *ELF3* (i), *GI* (j), *CO1* (k), *PHYA* (l), *PHYB* (m), *PHYC* (n), *CRY1* (o), *CRY2* (p) genes in association with the sampling times in three various environments (25°Cvern, 18°Cvern and 18°Cuv) (Error bars are based on standard errors among the 3 wheat genotypes). The grey rectangles indicate the duration of dark periods. *, **, *** denote significant relationships at the P ≤ 0.05, P ≤ 0.01 and P ≤ 0.001 probability levels, respectively. Two-week-old leaves were sampled every three hours for two consecutive days (48h). Relative gene expression values against the geometric means of three house-keeping genes are presented as the average of the two days studied.

Abbreviations: *CCA1* – *CIRCADIAN CLOCK-ASSOCIATED 1*, *CO1* – *CONSTANS 1*, *CRY1* – *CRYPTOCHROME 1*, *CRY2* – *CRYPTOCHROME 2*, *ELF3* – *EARLY FLOWERING 3*, *GI* – *GIGANTEA*, *LUX* – *ARRHYTHMO*, *PHYA* – *PHYTOCHROME A*, *PHYB* – *PHYTOCHROME B*, *PHYC* – *PHYTOCHROME C*, *PPD1* – *PHOTOPERIOD1*, *PRR95* – *PSEUDORESPONSE REGULATOR 95*, *TOC1* – *TIMING OF CAB EXPRESSION1*, *VRN1* – *VERNALIZATION1* (*APETALA1*), *VRN2* – *VERNALIZATION2*, *VRN3* – *VERNALIZATION3* (*FLOWERING LOCUS T*)

(a)


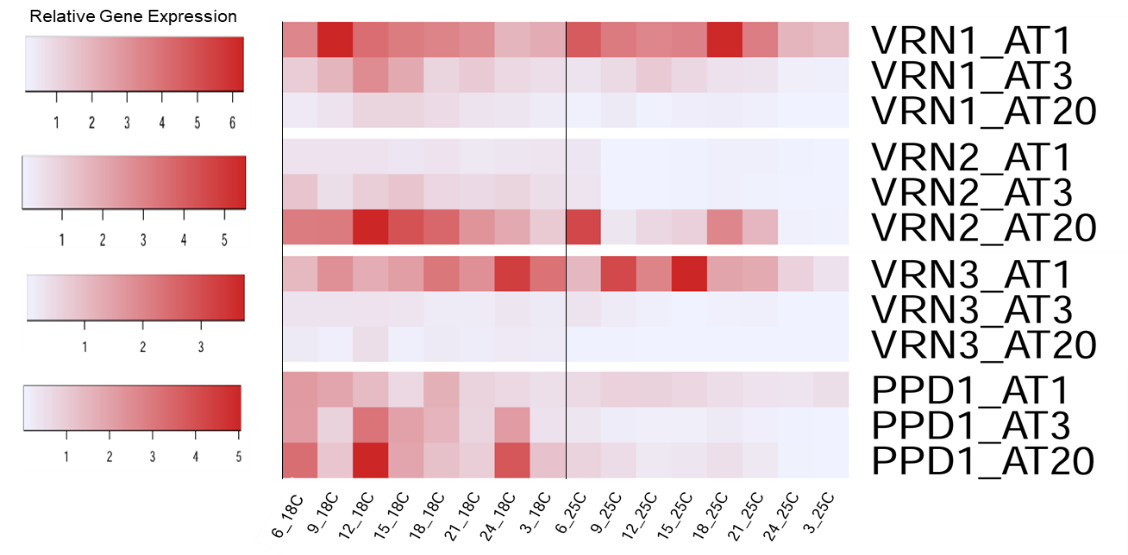


(b)


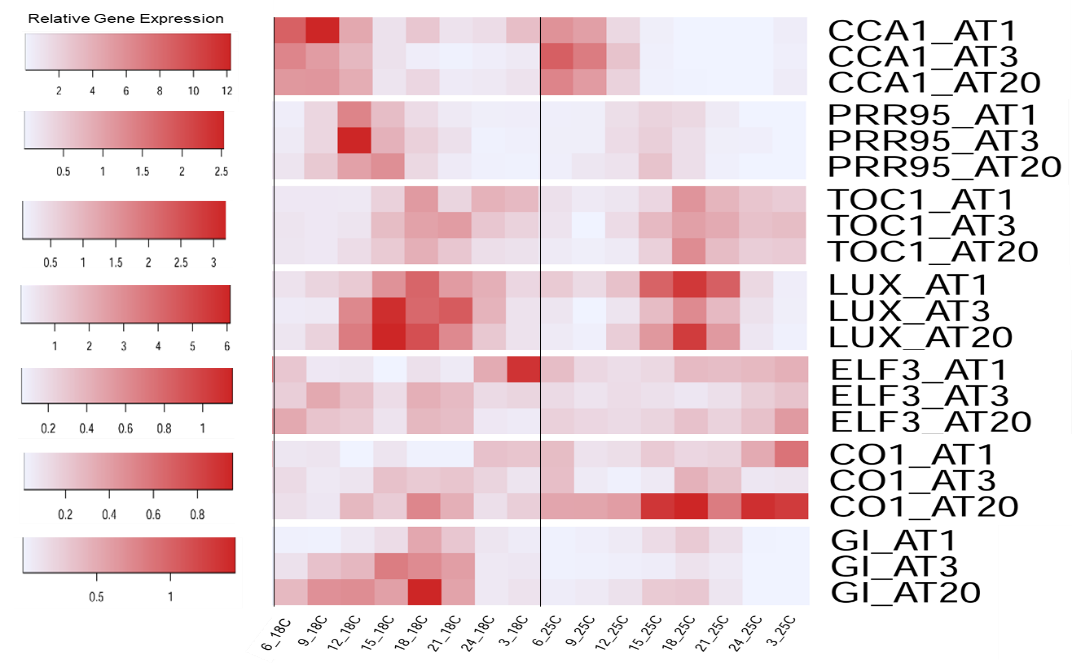


(c)


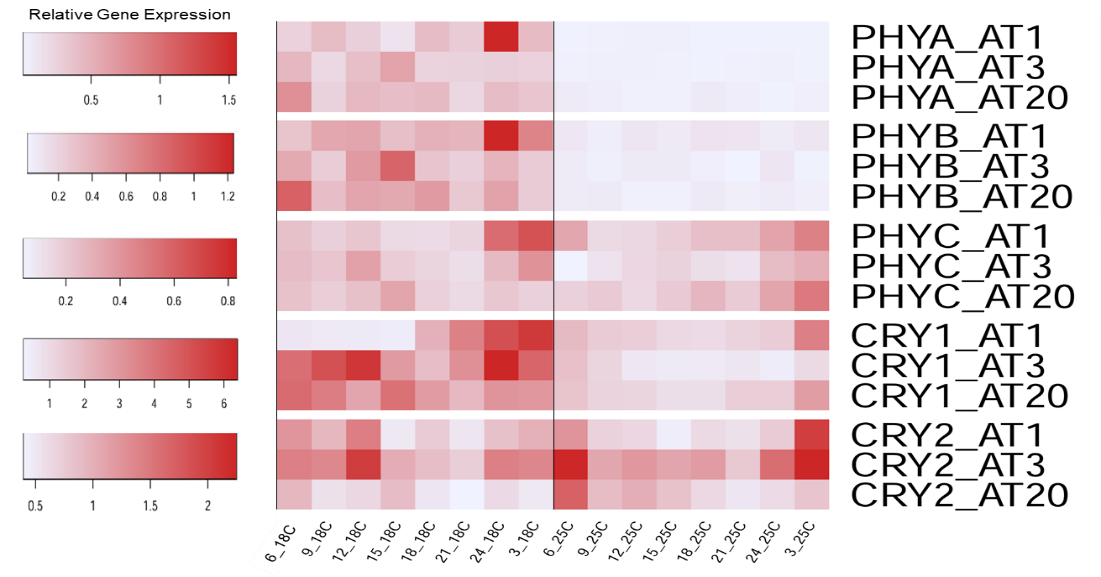


**Figure S4.** Heatmap of the daily average expressions of the plant developmental genes (a), the circadian clock genes (b) and the photoreceptor genes (c) in measured in the two vernalized treatments (18 °C and 25 °C) as compared to the percentage of average gene activities. The normalized counts of each gene represented by a color spectrum ranging from red (high expression) to grey (low expression).

Abbreviations: *CCA1* – *CIRCADIAN CLOCK-ASSOCIATED 1*, *CO1* – *CONSTANS 1*, *CRY1* – *CRYPTOCHROME 1*, *CRY2* – *CRYPTOCHROME 2*, *ELF3* – *EARLY FLOWERING 3*, *GI* – *GIGANTEA*, *LUX* – *ARRHYTHMO*, *PHYA* – *PHYTOCHROME A*, *PHYB* – *PHYTOCHROME B*, *PHYC* – *PHYTOCHROME C*, *PPD1* – *PHOTOPERIOD1*, *PRR95* – *PSEUDORESPONSE REGULATOR 95*, *TOC1* – *TIMING OF CAB EXPRESSION1*, *VRN1* – *VERNALIZATION1* (*APETALA1*), *VRN2* – *VERNALIZATION2*, *VRN3* – *VERNALIZATION3* (*FLOWERING LOCUS T*)

AT1: ‘Mv Toborzó’, from Hungary; AT3: ‘Tommi’, from Germany; AT20: ‘Charger’, from Great Britain
